# Fish welfare in a changing world: New developments and current challenges

**DOI:** 10.1111/jfb.70423

**Published:** 2026-04-21

**Authors:** Sonia Rey Planellas, João L. Saraiva, Eliane Gonçalves‐de‐Freitas, Pablo Arechavala‐Lopez, Bernice Bovenkerk, Michael Breen, Steven J. Cooke, Martin Føre, Lene Northwood, Lars Helge Stien, Sunil Kadri, Chris Noble, Jonatan Nilsson, Fernando Rodriguez, Cosme Salas, Peter Sandøe, Hans van de Vis

**Affiliations:** ^1^ Institute of Aquaculture, Faculty of Natural Sciences, University of Stirling Scotland UK; ^2^ IRTA, Research Institute for Food Technology, Welfare Unit Girona Spain; ^3^ Centre of Marine Sciences, CCMAR/CIMAR LA Faro Portugal; ^4^ Department of Biological Sciences, Institute of Bioscience Letters and Exact Science of the São Paulo State University (UNESP), and Aquaculture Center of UNESP São José do Rio Preto São Paulo Brazil; ^5^ Mediterranean Institute of Advanced Studies, IMEDEA‐CSIC/UIB Esporles Spain; ^6^ Social Sciences Group Philosophy Wageningen University and Research Wageningen Netherlands; ^7^ Fisheries Research Group, Institute of Marine Research Bergen Norway; ^8^ Department of Biology Carleton University Ottawa Ontario Canada; ^9^ Department of Engineering Cybernetics NTNU Trondheim Norway; ^10^ Independent law scholar; ^11^ Animal Welfare Research Group, Institute of Marine Research Bergen Norway; ^12^ UiT The Arctic University of Norway Tromsø Norway; ^13^ RINA, Research Institute for Environment and Livelihoods Charles Darwin University Brinkin Australia; ^14^ Division of Aquaculture Nofima Tromsø Norway; ^15^ Laboratory of Psychobiology, University of Sevilla Sevilla Spain; ^16^ Department of Food and Resource Economics and Department of Veterinary and Animal Sciences University of Copenhagen Copenhagen Denmark; ^17^ Wageningen Livestock Research Wageningen University & Research Wageningen Netherlands

**Keywords:** aquaculture, aquatic animals, fisheries, One Health/One Welfare, sustainability, welfare indicators

## Abstract

The welfare of non‐human animals is central to ethical discussions on animal use, with increasing attention to fish welfare across research, aquaria, aquaculture, and fisheries. This paper reviews current theoretical approaches to animal welfare and recent advances in defining and assessing fish welfare since the seminal paper by Huntingford et al. (2006; *J Fish Biol* **68**: 332–372), highlighting the growing role of cognitive and affective processes. It also includes the concept of positive welfare and some of the current research advances in this field. Methods for measuring, monitoring and assessing welfare via the utilisation of outcome‐ and input‐based indicators are outlined, ranging from practical operational tools to laboratory‐based measures. Welfare concerns in wild‐capture fisheries are examined in relation to stress, flesh quality and sustainability, including the welfare of released fish. Recent advances in fish neurobiology, cognition and pain perception are summarised, together with technological innovations that enhance welfare monitoring and management. The paper also explores the relationship between fish welfare, sustainability, public concerns and consumer demand, and legal and moral recognition across contexts, situating fish welfare within the ‘One Health’ and ‘One Welfare’ frameworks that link animal welfare, environmental stewardship and human well‐being. Ongoing challenges include climate change, cultural factors and the interpretation of fish sentience and cognition among others.

## 1 INTRODUCTION

### 1.1 Methods, aims and intended audience

This review paper aims to explore current fish welfare considerations, including their implications for research, aquaculture and husbandry, fisheries across sectors, aquariums and the impact of human activities on wild fish populations and human perception about fish welfare. It is not intended to be a systematic review given the broad scope of this topic. Nonetheless, it is anchored in literature and written by a diverse group of authors whose experience and perspectives were used to interpret the evidence. All authors reviewed all sections of the manuscript, ensuring that the paper is balanced and includes alternate interpretations and perspectives. This paper builds on the findings from the last Fisheries Society of the British Isles (FSBI) briefing paper in 2002 (FSBI, 2002) and the subsequent review paper by Huntingford et al. (2006), which identified critical knowledge gaps, uncertainties and contentious issues regarding the mental states of fish, pain perception, cognition and suffering. Nine key knowledge gaps were identified by Huntingford et al. (2006) that were fundamental to the whole concept of fish welfare and referred to a lack of information on (1) mental capabilities of fish and subjective states, (2) the behavioural needs of fish, (3) fish diseases and the links between stress and immune function, (4) welfare indicators, (5) the depth of existing information already available, (6) the ornamental trade and (7) commercial fisheries on fish welfare, (8) welfare knowledge on the range of fish species outside the salmonids and finally (9) clarifying the adverse effects of fish stressors on fish welfare. This review paper reflects on some of the advancements made through scientific research and new technologies regarding most of those areas of ignorance from the last 20 years (except for point 3, which would be too extensive for this review and where many specialised reviews on that subject have been already published).

These advancements have broadened our understanding of numerous fish welfare challenges and are helping to shape the development of new strategies for welfare improvement. While many topics require further discussion and study, this paper seeks to synthesise knowledge and identify some remaining gaps and uncertainties from the available research published on each of those fields. Furthermore, it addresses the question of what constitutes a good life for fish and how we can improve their welfare, recognising that fish play direct and indirect roles in supporting human culture, livelihoods, nutritional security, well‐being and other socio‐economic services.

The paper is aimed at a broad audience: those interested in keeping fish as pets, those who farm fish, those who catch fish (commercial, subsistence, recreational) as well as those who interact with fish in aquariums or zoological collections or as study subjects for research. We provide information that is applicable to all stakeholders in those sectors, including researchers and policy makers, on their judgement on fish welfare and what they can expect from the current advances and future challenges.

### 1.2 Positionality statement

Our authorship team includes a diverse suite of academic expertise such as neurophysiology, fisheries science, aquaculture, fish welfare, bioethics, legal studies, sustainability science, ethology, technology, production biology and veterinary science. Such a range of expertise is essential given the multidisciplinary nature of the issues under discussion, and they bring different backgrounds and opinions to the discussion, which are openly presented in the text. There is a lack of gender balance in the authorship composition. From the 17 co‐authors of the paper only four identify as women and rest identify as men, maybe a reflection of the field of research that has been mostly male dominated.

Authors are based in nine different countries, with most contributors based in Europe, with single authors in both Canada and Brazil and two in Australia. Regional variation enriches the diversity in cultural values, practices and social norms as it relates to animal (including fish) welfare (e.g. the United Kingdom and several countries in Europe have more cohesive and formalised animal welfare protections than other regions). Given that FSBI is a UK‐based organisation, and most of the participants are based in Europe, any policy implications of the work presented here need to acknowledge this limitation. We acknowledge that none of the authors are based in lower or lower‐middle income countries or identify as being Indigenous. Although we have attempted to find consensus, we have identified where areas of disagreement or multiple perspectives exist and where the evidence base is either weak (lack of replicated studies) or equivocal. The critical point is that all authors agree that fish welfare is important and there are many practical things that can be done to advance fish welfare in the context of research involving fish, in all fisheries sectors, in fish husbandry for aquaculture or the aquarium industry, for fish conservation and for the general public who keep fish as a pastime.

### 1.3 Why is fish welfare important?

Both farmed and wild‐caught fish play an important role in many people's diets across the globe (Holmlund & Hammer, 1999; Lynch et al., 2016). Fish are also widely used for various forms of research: fisheries, aquaculture aimed at securing livelihoods and healthy foods to innumerable people, and biomedical research aimed at preventing, curing or alleviating human diseases and other health problems (Lynch et al., 2016). They are aesthetically fascinating when on display in public or private aquariums or ponds, or when divers and swimmers meet them in various, often touristic, settings. According to statistics compiled by the European pet food industry there were about 23 million privately owned ‘pet’ fish in Europe in 2022 (FEDIAF, 2022). In addition, according to statistics compiled by the American Veterinary Medicine Association, around 3% of US households keep fish as companion animals (AVMA, 2018). A significant group of pastime and sports anglers also interact with fish (Arlinghaus et al., 2015). Finally, thriving populations of wild fish are an important indicator of the state of the environment.

## 2 THE NEUROBIOLOGY OF FISH WELFARE

Comparative research on the brain and behaviour has long been influenced by the belief that vertebrate evolution follows a linear path, progressing from ‘inferior’ to ‘superior’ forms, culminating in humans (Striedter & Northcutt, 2019). This perspective suggests that fish possess simple neural circuits that only support basic, reflexive behaviours, lacking the complex brain structures necessary for advanced cognition or any degree of sentience. However, recent decades of comparative neurobiological and psychobiological research have challenged this view, revealing that the behaviour and brain organisation of teleost fishes are far more intricate and sophisticated than previously assumed. Despite the remarkable diversity and specialised adaptations seen across different species, research has shown that the brains of all vertebrates, including fish, follow a shared organisational blueprint inherited from a common evolutionary ancestor. This shared schema, or *Bauplan*, encompasses similarities in brain subdivisions, patterns of early regulatory gene expression, molecular and neurochemical distributions, sensory and motor pathways, and intrinsic neural circuits, as well as mechanisms for cognitive and emotional processing (Wullimann & Vernier, 2009). Furthermore, the persistent belief that fish possess small and relatively simple brains—an idea still perpetuated uncritically in some academic circles—is firmly contradicted by comparative evidence. In fact, the brains of certain fish species exhibit remarkable sophistication and cytoarchitectural differentiation that represent milestones in the evolution of the complexity of the vertebrate brain. For instance, large‐brained elasmobranchs, basal osteoglossomorph teleosts and some more derived ostariophysans and percomorph euteleosts show pronounced hypertrophy of specific brain regions and possess encephalization quotients comparable to those of birds and mammals (Striedter & Northcutt, 2019). This challenges long‐standing assumptions about the limitations of fish neural complexity and behavioural capacities, rooted in the now‐discredited ‘Scala naturae’ concept of vertebrate brain evolution.

### 2.1 Brainstem and body state representation in fish

**FIGURE 1:**
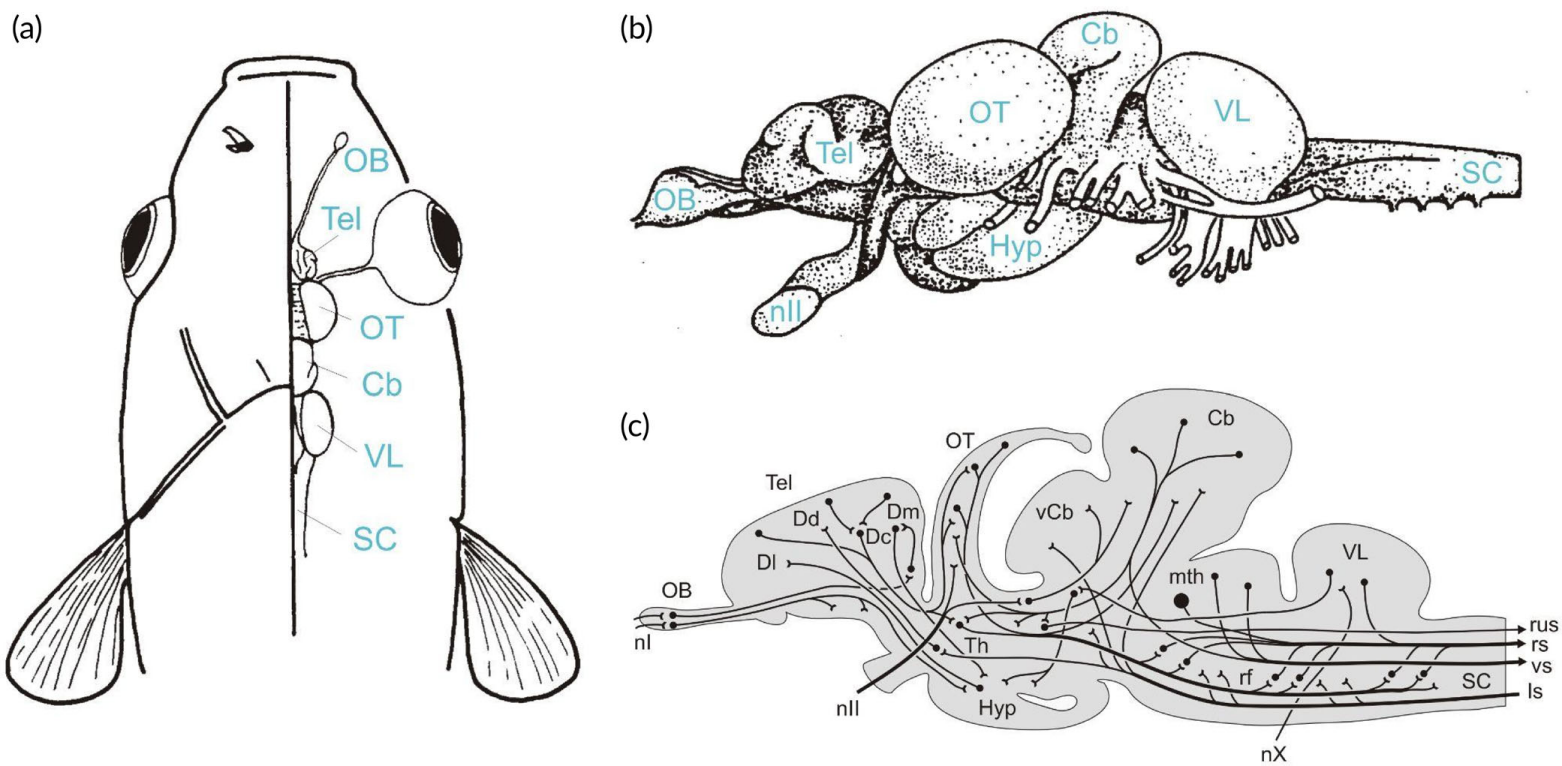
The brain of a teleost fish. (a) Dorsal view of the goldfish brain. (b) Lateral view of the goldfish brain showing the main brain subdivisions. (c) Major circuits of the teleost fish brain. Note that the basic pattern of connectivity is highly similar to that observed in land vertebrates. Cb, cerebellum; Dc, area dorsalis telencephali pars centralis; Dd, area dorsalis telencephali pars dorsalis; Dl, area dorsalis telencephali pars lateralis; Dm, area dorsalis telencephali pars medialis; Hyp, hypothalamic lobe; ls, lemniscus spinalis; mth, Mauthner cells; nI, olfactory nerve; nII, optic nerve; nX, vagal nerve; OB, olfactory bulb; OT, optic tectum; SC, spinal cord; Th, thalamus; rs, fasciculus reticulo‐spinalis; rf, reticular formation; rus, tractus rubro‐spinalis; v, ventricle; vCb, valvula cerebellum; VL, vagal lobe; vs., fasciculus vestibulo‐spinalis. Modified from Broglio et al. (2003).

The peripheral nervous system, spinal cord, hindbrain, midbrain and diencephalon in fish show striking anatomical and functional similarities to those of mammals and other vertebrates, indicating that essential mechanisms in neural development have been conserved throughout vertebrate evolution. Key structures within the mammalian caudal neuroaxis—such as the area postrema, nucleus of the solitary tract, parabrachial nucleus, vagal and trigeminal nuclei, reticular formation, periaqueductal grey, cerebellum, optic tectum and hypothalamus—have counterparts in the fish brain (Ishikawa et al., 2022). Such brain regions, like those in other vertebrates, play crucial roles in continuously monitoring the body's internal state and generating motor and visceral responses (Marcus et al., 2014; Saper & Lowell, 2014). This includes chemosensation, thermal and nociceptive perception, balance, self‐movement, force and body position sensing. Additionally, they are involved in a wide range of viscerosensory and visceromotor responses, such as cardiovascular, respiratory and gastrointestinal functions, as well as in generating drives and valenced emotional behaviours, including reward, hunger, sleep, aggression and escape responses. Prominent neurobiological theories of sentience propose that body‐state mapping by brainstem and diencephalic mechanisms may suffice for the emergence of awareness in animals (e.g. Damasio, 2010; Feinberg & Mallatt, 2013; Merker, 2007; Panksepp, 2005). Given the remarkable conservation of neuroanatomical and neurofunctional features across fish and terrestrial vertebrates, it is reasonable to argue, following the principle of parsimony, that these brain structures may play similar roles in generating body awareness and sentience in both fish and land vertebrates. See Figure 1 to follow the brain structures in the text.

### 2.2 Cerebellum and optic tectum

Within the hindbrain and midbrain, structures such as the cerebellum and optic tectum are essential not only for motor control but also for cognitive and emotional functions. The cerebellum in fish shares fundamental organisational neuroanatomical features with that of other vertebrates, contributing, much like in mammals, to perception and attention modulation, learning and memory, and emotional conditioning (Durán et al., 2010; Rodríguez et al., 2005). This suggests that cognitive and emotional functions associated with the cerebellum may have emerged early in vertebrate evolution and have been conserved throughout phylogeny. The optic tectum is another critical midbrain structure involved in sensory processing and response generation (Bianco & Engert, 2015; Northmore, 2011; Salas et al., 1997). Its distinct layered structure receives topographically ordered visual inputs from the retina and integrates them with other sensory modalities, such as auditory, somatosensory and electrosensory inputs. This brain structure is positioned at the top of a neural hierarchy that processes sensory information and extracts increasingly abstract features of stimuli, merging sensory modalities to create a unified perceptual world and to guide motor responses. Thus, the optic tectum in fish plays a pivotal role in selective attention, goal‐directed behaviour and perceptual decision‐making (Ben‐Tov et al., 2015; Bianco & Engert, 2015; Northmore, 2011). Therefore, the deeper layers of the optic tectum contain a spatially organised motor map that aligns with sensory maps and projects to the brainstem motor centres, enabling the programming and generation of orientation responses. In fact, the optic tectum constructs an internal model that continuously tracks the fish's body and gaze position, facilitating predictive and planned movement by simulating the current and future states of body parts. This internal model provides a body‐centred frame of reference for sensory‐motor transformations and for generating egocentrically referenced actions in space. Such a midbrain structure creates a unified spatial model of the fish's body in its environment, potentially generating awareness of its position and actions in the surrounding. Theories of awareness suggest that linking valenced body‐state mapping with sensory inputs from the outside world, combined with a neural simulation of the agent's body position in space in egocentric coordinates, may provide the foundation for generating a self‐centred, or first‐person, perspective and, consequently, subjective experience (Butler, 2012; Gazzaniga et al., 2019; Graziano et al., 2020; Panksepp & Biven, 2012).

### 2.3 Telencephalic pallium and higher‐order memory representations in fish

Fish can represent space in ways that extend beyond self‐centred frameworks; like land vertebrates, they can also use allocentric, map‐like spatial memory representations of their surroundings (Rodríguez et al., 2021; Salas et al., 2017). These ‘external world‐centred’ spatial representations encode the objective spatial relationships between multiple landmarks, along with topographic features and the geometry of environmental boundaries, allowing animals to position themselves objectively within their surroundings (Rodríguez et al., 2021). Such relational spatial representations provide significant behavioural flexibility, allowing for actions such as taking shortcuts or successfully navigating from new starting points (Rodríguez et al., 2021; Salas et al., 2017). Furthermore, neurobehavioural studies indicate that the dorsolateral area of the teleost fish telencephalic pallium, a structure likely homologous to the mammalian hippocampus, plays a crucial role in map‐like, relational spatial memories. Controlled experiments have shown that allocentric navigation selectively activates the teleost hippocampal pallium and that damage to this structure impairs the ability to use map‐like spatial memories (Ocaña et al., 2017; Rodríguez et al., 2002, 2021). Importantly, the recent discovery of ‘place cells’ in the zebrafish (*Danio rerio*) telencephalic pallium (Yang et al., 2024) further indicates that the intimate hippocampal mechanisms for spatial navigation and relational memory are similar across fish, birds and mammals, suggesting an ancient evolutionary origin for these neural mechanisms and the conservation of their essential features throughout the evolution of various vertebrate lineages. It should be noted that allocentric representations not only encode the external environment in a stable, viewer‐independent manner, but also integrate the subject's own position within that spatial framework. The capacity to represent ‘otherness’ and to perceive oneself within the environment, akin to a ‘third person’ perspective, aligns with theories that highlight ‘allocentricity’ as a crucial criterion for recognising awareness in non‐human animals (Behrendt, 2013; Jeffery & Rovelli, 2020; Seth et al., 2005; Woodruff, 2018).

Furthermore, the hippocampal pallium of teleost fish plays an essential role not only in spatial memory but also in recalling temporally organised experiences and in associating items across various cognitive dimensions. Like the hippocampus in mammals, it is selectively involved in trace classical conditioning and in encoding sequences of experienced items, thus being essential for linking events encountered in separate episodes (Gómez et al., 2022; Rodríguez‐Expósito et al., 2017). This recent evidence suggests that the cognitive representations supported by the hippocampal homologue in teleost fish share several fundamental functional features that define semantic and episodic memory in mammals and birds. In addition, the teleost fish hippocampal pallium also supports transitive inference, enabling prospective decisions through inferences about indirectly related past experiences (Sotelo‐Parrilla et al., 2025). Consequently, as in mammals and birds, the teleost fish hippocampal‐dependent relational memory system supports the flexible expression of learned information, allowing generation of expectations about future outcomes and formulation of future scenarios that can effectively guide fish behaviour. In mammals, an intimate connection between episodic memory and awareness has often been suggested, and the hippocampus and its associated neural circuits have been proposed as key substrates supporting awareness (Behrendt, 2013; Byrne et al., 2007; Squire et al., 2004; van den Bos, 2020). The allocentric nature of conscious experience, the embedding of personal experiences within an external spatial and temporal framework, and the binding of multiple dimensions of experience into a unified spatiotemporal representation could indicate a role of the hippocampus in the generation of the subjective experience of events that unfold across space and time. In fish, the internal representations supported by the hippocampal pallium may provide substantial adaptive advantages by enabling the integration of experiences across extended time and space scales, constructing predictive models of the environment that allow for anticipatory responses, and generating attention schemas that facilitate the focused control of perceptual and cognitive processing through covert attention.

### 2.4 Neurobiology of pain in fish

In recent decades, a growing body of psychobiological and neurobiological comparative research has defied the traditional view that higher cognitive and emotional functions are exclusive to mammals and birds, revealing that fish exhibit complex and nuanced emotional behaviour (Brown et al., 2011; Salas et al., 2006). Affective states are not merely linked to basic reflexes or instinctive behaviours; they generate positively or negatively valenced experiences that enable the self‐referenced categorisation of encountered events. By prioritising significant experiences and relationships during memory encoding, affective states create value maps of environmental events that serve as predictive models, motivating and guiding future actions. In this way, affective processing not only generates motivation that drives animal actions but also facilitates anticipatory behaviour and efficient decision‐making, ultimately enhancing the fish's ability to survive and adapt to its environment. Furthermore, experimental evidence indicates that affective states in fish rely on distinct neural systems that extend beyond the brainstem and hypothalamic circuits to include structures homologous to the cerebral cortex of mammals (Maximino et al., 2013; Salas et al., 2006).

A crucial aspect of emotional processing with significant implications for fish welfare is their capacity to experience pain. Pain is an aversive somatosensory sensation that signals tissue damage or potential harm, triggering physiological and behavioural responses aimed at avoiding, minimising or preventing further injury or promoting recovery. Experiencing pain is a complex psychobiological phenomenon involving not only sensory components but also attentional, affective, memory and cognitive dimensions. In some teleost fish species, nociceptors associated with C and A‐delta fibres have been identified, including mechanothermal, mechanochemical and slow‐adapting polymodal types (Ashley et al., 2007; Matthews & Wickelgren, 1978; Sneddon, 2002, 2003). These nociceptors respond to intense mechanical pressure, extreme heat and harmful chemicals, exhibiting physiological response properties similar to those observed in mammals. The nociceptive signals generated by these receptors in fish are transmitted via spinal and trigeminal tracts to a broad network of neural centres, including the brainstem, cerebellum, diencephalon and telencephalon (Dunlop & Laming, 2005; Luiten, 1975; Malafoglia et al., 2013; Nieuwenhuys & Pouwels, 1983; Nordgreen et al., 2007; Puzdrowski, 1988; Reilly et al., 2008; Sneddon, 2003). This network exhibits a neuroanatomical organisation like that of tetrapods, suggesting that pain processing in fish goes beyond simple nociceptive (‘reflexive’) responses. Instead, it represents a complex neurobiological and psychological phenomenon arising from the interaction between afferent sensory information and the activity of higher‐order attentional, affective, mnemonic and cognitive brain circuits.

When subjected to noxious stimuli, such as electric shocks, skin pinches or subcutaneous injections of algogenic substances such acetic acid, formalin or histamine, fish exhibit various behavioural and physiological changes suggesting an aversive experience (e.g. defensive and escape responses, increased ventilation rates, changes in cardiac activity, elevated levels of stress hormones, reduced activity, altered posture, the suspension of feeding and altered social or antipredator behaviour) (Chandroo et al., 2004; Segner, 2012; Sneddon, 2023). Moreover, these behavioural and physiological alterations are significantly reduced, in a dose‐dependent manner, when fish are administered pain‐relieving drugs, such as lidocaine, morphine and other opioid agonists, or cannabinoids (Sneddon, 2023), indicating the presence of complex analgesic and pain modulation mechanisms.

Additional behavioural data shows that noxious events trigger not only immediate, automatic nocifensive responses but also lead to long‐term motivational shifts and delayed behavioural changes. These adaptations help fish avoid or reduce future encounters with painful stimuli by influencing memory‐based decision‐making. Examples include hedonic value‐based decisions and behavioural cost–benefit trade‐offs, developing conditioned place preferences linked to pain relief, or dynamically and flexibly modulating coping with painful events by competing motivations such as hunger, access to opioids, social factors (e.g. the presence of conspecifics) or familiarity with their environment (Chandroo et al., 2004; Segner, 2012).

Further evidence supporting the notion that the behavioural responses of fish to noxious stimuli represent pain perception rather than mere nociception (a simple reflex reaction) comes from studies on classical fear conditioning and operant conditioned avoidance learning (Broglio et al., 2005). In these studies, a previously neutral stimulus (e.g. light or sound) becomes a conditioned aversive stimulus when it predicts the future occurrence of a painful experience (e.g. an electric shock). After conditioning, the formerly neutral stimulus evokes a fear response that is behaviourally and physiologically similar to the unconditioned pain response caused by the noxious stimuli. Thus, the fear response reflects the fish's expectancy of a future painful experience. While both fear and pain are unpleasant affective states, fear arises from the threat of an impending noxious event, suggesting not only the awareness of painful experiences but also the ability to anticipate them. This anticipatory capability requires complex affective and cognitive representation and processing mechanisms, far beyond simple reflexive responses that rely only on lower brain centres, such as the spinal cord and brainstem. Furthermore, neurobehavioural evidence demonstrates that, like in mammals, these affective and cognitive capabilities depend on fish telencephalic structures, particularly the dorsomedial area (Dm) of the pallium, a region that could be homologous to the basolateral amygdala and other areas of the limbic cortex in mammals (Lal et al., 2018; Portavella et al., 2004). Moreover, the Dm area of the fish pallium appears to play a specific role in modulating pain perception (Wolkers et al., 2015, 2017). Some have questioned whether fish can feel pain, arguing that they lack telencephalic structures equivalent to the cerebral cortex of mammals, a brain region they consider essential for conscious pain perception (Arlinghaus et al., 2007; Browman et al., 2019; Key, 2015, 2016; Rose et al., 2014). However, neuroanatomical, neurochemical, neurodevelopmental, gene expression and neurofunctional studies indicate that the telencephalon of all vertebrates, including fish, is organised into two main regions: the pallium and the subpallium (Salas et al., 2006; Woodruff, 2017). The pallium, which is the homologue to the cerebral cortex of mammals, consists of at least four primary subdivisions consistently found across all vertebrate lineages, including fish (Puelles, 2017; Striedter & Northcutt, 2021). Like the mammalian cerebral cortex, the telencephalic pallium in fish is involved in sensory processing, relational memory and higher‐order emotional and cognitive functions, and therefore it could also play a central role in pain experience in fish. Although we have (briefly) summarised the existing evidence base regarding pain in fish, knowledge remains relatively limited and very few studies have been replicated across species or other contexts, making it difficult to generalise. However, the discussion of the existence of pain in fish plays a central role in the history and development of fish welfare, as we will see in the next sections.

## 3 DEFINITION(S) OF WELFARE FOR FISH

### 3.1 Why it is important to define welfare for fish

The scientific approach to farm animal welfare was established in the last half of the 20th century (Broom, 2011), yet the incorporation of fish into animal welfare science is much more recent. It was not until the 21st century that fish scientists began to address the issue cohesively and from a research perspective (Kristiansen & Bracke, 2020). Given its relatively young age, it is not surprising that fish welfare still is a topic area with disagreements, diverging views, strong opinions and miscommunication between stakeholders.

Furthermore, there is lack of consensus between differing stakeholders (e.g. farmers, fishers, consumers, retailers, non‐governmental organisations [NGOs], scientists, policymakers) on the definition of ‘animal welfare’, who can have different interpretations of the term. It should be acknowledged that animal (fish) welfare not only involves applications of fundamental science but also has an ethical and philosophical dimension. It is, however, essential to be transparent about underlying assumptions so that a constructive dialogue can address key questions, such as how we can measure, monitor, ensure and regulate welfare for fish.

### 3.2 Fundamental philosophical approaches to welfare

Any attempt to define fish welfare will rely on the author's underlying assumptions and approaches to the concept of welfare as it applies to both humans and non‐human animals. Sandøe (1999) and Appleby and Sandøe (2002) divide this up into three competing approaches: hedonism, preference‐satisfaction theory and perfectionism. The focus of hedonistic theories is on the balance between the individual's positive over negative experiences. In these perspectives, the subject's cognitive appraisal of the world and its derived mental states are the main base for welfare. From hedonistic approaches, welfare is seen not merely as something that is provided (i.e. a passive view of the subject), but also as something experienced: subjects are agents in their world and capable of experiencing not only negative states but also pleasures and overall positive welfare (Rault et al., 2025). The main assumptions from hedonistic approaches are that a wide range of sentient beings, including fishes, have the capacity to feel things and to have a mental experience of the world. Ultimately, this mental/cognitive balance between positive and negative experiences is what matters for welfare (Spruijt et al., 2001).

The focus of preference‐satisfaction theories is that welfare is determined by the satisfaction of preferences and desires, regardless of the outcome. Welfare is enhanced when one's preferences are met, regardless of whether those preferences lead to better overall outcomes (happiness, health, fitness) or not (Baber, 2011).

Under perfectionist approaches, welfare emerges as a biological performance challenge. Although mental experiences may matter, the exercise of functions assume higher importance. The main assumption of perfectionist approaches is that welfare is linked to the realisation of natural potentials and not necessarily merely to feeling well while doing it (Appleby & Sandøe, 2002).

Another way to consider fundamental philosophical approaches to specifically animal welfare is to divide them into function‐, nature‐ and feeling‐based approaches (Fraser, 2009). Function‐based approaches focus on the health and physiology aspects of animal welfare, that is if an animal can adjust to its environment, such that all its biological functions are working effectively. Nature‐based approaches assume that each species has an inherent biological nature and that the ability to express it (particularly to express a repertoire of natural behaviours) is essential for good welfare and overlap with perfectionism as described above. Feelings‐based approaches focus on the animal's mental state, how it experiences its life and overlap with hedonism, as described above. These three approaches will in practice often overlap, in that, for example, an animal's mental state depends both on its behavioural freedom and its physical state, and an animal's mental state can again influence its physiology and behaviour.

### 3.3 Key frameworks to operationalise animal welfare

#### 3.3.1 The five freedoms

One of the earliest frameworks that attempts to operationalise the idea of animal welfare dates to 1979, when the Farm Welfare Council of the UK published a list of five fundamental freedoms for farm animals (freedom from hunger, discomfort, pain, fear and to express natural behaviour), later revised several times. Current considerations of welfare in farmed animals often draw heavily on this concept, which forms the basis of numerous recommendations and legislations worldwide and is still extensively employed for academic, educational and veterinary purposes, with great practical utility (McCulloch, 2013). The five freedoms concept is nevertheless open to criticism. For example, it has been argued that it incorrectly implies that captive animals are passive, rather than engaging actively with their environment (Ohl & van der Staay, 2012). In addition, and understandably at the time in 1979, the emphasis was very much on protecting animals from negative experiences, epitomising the view that ‘free from harm equals good’. Even the freedom to express natural behaviour was widely understood to mean absence of frustration of behavioural needs. Recent approaches expand the welfare concepts to conceive the possibility of positive experiences in farm animals, including fish (Rault et al., 2025), which is explained further in the positive welfare section.

#### 3.3.2 Broom's definition of welfare

Following a version of the perfectionist approach and aiming to create an operational definition where welfare could be somehow quantified, Donald Broom proposed in 1986 that welfare can be seen as ‘the state of the animal as it attempts to cope with its environment’ (Broom, 1986, 1991). This framework proposes that (i) welfare is a characteristic of an animal, not something that is given to it, (ii) welfare will vary from very bad to very good, that is, along a welfare continuum, (iii) welfare can be measured objectively, (iv) measures of failure and difficulty to cope with the environment give information about how poor the welfare is, (v) knowledge on the biology and life history of an animal provide essential information about suitable rearing conditions, but direct measurements of the state of the animal must also be used to assess its welfare, and (vi) coping mechanisms may vary among different species, and there are several consequences of failure to cope. Therefore, any one of a variety of measures can indicate that welfare is bad, and the fact that one measure, such as growth, is normal does not mean that welfare is good (Broom, 1991). Although in the initial version mental states were not obviously considered, later revisions of this proposal explicitly included animal feelings (Broom, 2016). This definition of welfare and the concurrent framework laid some of the theoretical foundation for the development of early operational welfare indicators (OWIs) for farmed fish (e.g. Branson, 2008). We will discuss OWIs further in the next section on how to measure welfare.

#### 3.3.3 The five domains model

The five domains model proposes that nutrition, physical environment, health and behavioural interactions must be considered as domains that contribute to a fifth domain, the mental state of the animal. Thus, this framework is clearly in line with the hedonist approach described above. The first three domains focus attention on internal processes that have nutritional, environmental and health origins. In contrast, the latest iteration of the fourth domain focuses on the animal actively, intentionally and purposefully pursuing definite goals in their behavioural interactions with the environment and other animals (including humans). The fifth domain enables an ultimate assessment of the overall welfare state of the animals, understood in terms of what they were likely to experience subjectively (Mellor et al., 2020; Mellor & Reid, 1994). The initial model did not account for positive experiences, but later revisions do so (Mellor et al., 2020; Mellor & Beausoleil, 2015).

#### 3.3.4 The ‘needs’ concept

The ‘needs’ concept comprises all needs that affect an animal's welfare state by generating positive (pleasure) or neutral feelings when satisfied and aversive feelings when frustrated (Bracke et al., 1999; Kristiansen & Bracke, 2020). It defines an individual's welfare state as the animal's affective state, its sum of feelings, that is, the quality of life as perceived by the animal itself (Bracke et al., 1999). Perhaps inspired by the five freedoms and the five domains, five overarching welfare needs are proposed for farmed fish (Stien et al., 2020): an appropriate water environment, adequate nutrition, good health, behavioural freedom and safety. The need for an appropriate water environment includes all needs related to the quality and constituents of the ambient rearing water that are necessary to fulfil bodily functions, such as respiration, osmoregulation and thermoregulation. Water quality is obviously essential for the welfare of aquatic animals, with significant impacts on many biological functions (Zhang et al., 2025). Nutritional needs relate to feed and nutrition. Fulfilling the need for food is essential for fish growth, physiological functioning and health. Good health is a major welfare need and has for humans been defined by the World Health Organisation as ‘a state of complete physical, mental and social well‐being and not merely the absence of disease or infirmity’ at the International Health Conference in 1946 (https://iris.who.int/handle/10665/85573). Fulfilment of this need not only requires absence or a low level of malformations, diseases, parasites and injuries, but also a well‐functioning physiology (brain–body), fitness and vigour. Behavioural needs include being able to control bodily movement, avoid collisions and move as intended, but also to live in accordance with natural tendencies/inclinations, the possibility of choice and being able to search and gain access to resources (foraging), social contact (in social species), migration and reproduction (at the relevant life stages), favourable environmental conditions (light, temperature, etc.) and rest (Boltaña et al., 2013; Mellor, 2016; Noble et al., 2018; Rey et al., 2015; Stien et al., 2020). Finally, safety relates to the need to protect the individual's bodily integrity. This means that fishes can protect their bodies from injury by escaping from predators, other organisms and objects perceived as threatening. They can also learn to avoid objects and environments associated with previous fearful, harmful or painful experiences, as explained at the fish neurobiology section. The proposal made by the needs concept assumes that how an animal experiences its life is not directly assessable for us, but by using available knowledge about the biology of the species we can identify observable parameters that give an indication of how well the different welfare needs are fulfilled, and from this derive an evaluation of the animal's welfare state (Bracke et al., 1999; Kristiansen & Bracke, 2020). This approach also considers the possibility of both negative and positive welfare states.

### 3.4 Positive welfare

Positive welfare states have been proposed for fishes (Fife‐Cook & Franks, 2019) based on the premise that fish (as other vertebrates) are equipped to seek positive affective states and that these are indeed adaptive (Fraser & Duncan, 1998; Romanes, 1883) as previously seen. According to this view, knowledge of fish biology and behaviour should allow the identification of such positive affective states. Examples of behaviours that fish may perform because they feel good include play (Burghardt, 2015), preferential attachment, aka ‘friendship’ (Heathcote et al., 2017), social buffering of fear aka ‘seeking comfort’ (Faustino et al., 2017), displays of motivation and preference (Galhardo et al., 2011; Maia et al., 2024; Maia & Volpato, 2017; Rey et al., 2015) or free‐choice exploration (Graham et al., 2018). Specific tests to measure affective states such as cognitive/judgement bias have been developed for fish (Espigares et al., 2022). Interestingly, a candidate to provide the conditions for the emergence of positive welfare in fish can be the application of environmental enrichment (Kleiber et al., 2023). A recent paper aiming to consolidate terms and definitions for the study of positive animal welfare (PAW) in animals proposed the following definition: ‘PAW can be defined as the animal flourishing through the experience of predominantly positive mental states and the development of competence and resilience. PAW goes beyond ensuring good physical health and the prevention and alleviation of suffering. It encompasses animals experiencing positive mental states resulting from rewarding experiences, including having choices and opportunities to actively pursue goals and achieve desired outcomes’ (Rault et al., 2025).

It becomes apparent that current frameworks for understanding animal welfare seem to converge towards mental and/or affective components in their welfare considerations. Emotion and affect in combination with advanced cognitive abilities confer on humans and many non‐human animals the capacity to compliment and/or override immediate reflexes to stimuli and so allow a large degree of flexibility in behaviour. In fishes, existing evidence suggests that the brain–behaviour relationships are not fundamentally different from those observed in mammals. Furthermore, data also seem to show that behaviour patterns related to emotion and cognition vary between fish species, as well within fish species, related to, for example, sex and the life‐history stage of the fish. From a welfare perspective, knowledge of such variability will potentially help us to design optimal living conditions for fish species kept by humans (Braithwaite et al., 2013).

### 3.5 Ethics

Finding out about the welfare consequences of different ways of treating fish does not by itself tell us how to act. To get to this an ethical frame or principle is required. Different ‘schools’ of ethics can be applied to questions around fish welfare (see Huntingford et al., 2006). Whereas utilitarians argue we should weigh potential harms against benefits in our dealings with fish, animal rights proponents take a more principled approach and argue that fish have a right to good welfare.

Relational ethics is a relatively new theory that differentiates between domesticated animals, towards whom we have a positive duty to look after their welfare, and wild animals, towards whom we only have the duty to leave them alone as much as possible. Domesticating or capturing animals creates a moral commitment towards them (Palmer, 2010). In contrast, according to some animal ethicists we also have a duty to relieve the suffering of animals in the wild, even if this suffering was not caused by humans (Horta & Teran, 2023). Those favouring this view think we should only take the affective states of animals into account in our dealings with them. Yet other theories, in particular environmental ethics, argue our first duty is to protect natural biodiversity and that we should allow fish to lead natural lives. In the following text we present several issues around fish welfare that have become the subject of debate recently and relate them to the above‐mentioned ethical views.

When weighing the interests of animals against those of humans, the purpose for which animals are used is widely considered morally relevant. For example, all ethical schools suggest that when people rely on fish for their survival or livelihood, they have a stronger claim to kill fish or harm their welfare than when they do so for entertainment or recreation. However, moral judgements will always be context dependent. This can be seen in recent debates on angling (Bovenkerk & Meijboom, 2020; Elder, 2018; Muir et al., 2013) and aquaria (Maceda‐Veiga et al., 2016a; Zoo and Aquarium Visitors’ Wildlife Values and Ethics Orientations, 2023), which many people think lack a moral justification while others see as a legitimate interaction with or way of preserving wild nature. Another ethical debate concerns the question of whether humans are allowed to kill fish for consumption. Certain theorists argue that fish do not have a concept of death and therefore have no preference to stay alive. In contrast, from an animal rights perspective it is argued that animals have a right not to be harmed, and therefore we need to ask whether killing them constitutes a harm. Some argue that as fish are sentient creatures who strive for certain goods, it is therefore morally harmful to kill them prematurely, as this takes away all their future possibilities for experiencing those goods (Bovenkerk & Braithwaite, 2016). Others argue that death would only constitute a harm for fish if they were self‐conscious, as they only then would have ‘categorical desires’, giving them a reason to want to continue living (Cigman, 1981). For those theories that do not consider death a harm, it is still relevant to consider the harm to their welfare during fishing and killing. In fisheries these debates are particularly important (see the sections on fisheries and welfare and on sustainability).

There has been debate over whether genetic modification techniques should be used in aquaculture to increase growth rate, control the maturation of fish, create resistance against parasites and make fish sterile to avoid the potential problem of escaped fish interbreeding with wild conspecifics. From a utilitarian point of view, the main moral concern relates to potential welfare problems. Animal rights theorists have additional concerns about the violation of the fish’ integrity and their instrumentalization (Winther et al., 2024). For environmental ethicists, the value of wildness is at stake. The use of digital technologies and precision fish farming (Føre et al., 2018) also raise moral concerns, as these could lead to alienation between the farmer and their fish, which is an issue for relational ethics as well as the potential discrimination against small farmers unable to access these new technologies for economic reasons. Rights theorists, furthermore, point out the risk of objectifying fish, and all theories are worried about potential severe welfare problems in case of technological failure.

In the context of recreational fishing there is also a discussion about the possibility of ‘ethical angling’ and the merits of catch and release versus catch and kill (Holmes, 2020). Many consider fishing with live bait to be one of the cruellest forms of fishing and an increasing number of countries have banned it.

While proponents of different ethical theories disagree on many of these issues, there is broad support for improving fish welfare. Achieving this requires a pragmatic approach and looking for an ‘overlapping consensus’ on issues (Rawls, 2002) and sometimes compromises need to be made. Whether such compromises are viewed as acceptable depends on one's ethical stance. Utilitarians regard all reforms that genuinely improve welfare as a step in the right direction, while some adherents of animal rights and relational approaches will oppose any less than ideal treatment of animals. Yet others will endorse a form of what has been labelled ‘non‐ideal’ theory to make room for compromises, accepting that in situations where it is not possible to reach agreement on radical reform, we should still work to improve animal welfare (Garner, 2013).

### 3.6 Critical anthropomorphism

Anthropomorphism, which means the application of human‐like attributes such as feelings and intentions to objects and animals, has been criticised by many. Some are concerned that it leads to misrepresentation of the similarities between humans and other animals, while others are concerned that it elevates animals to human‐like levels, making activities such as fishing unethical, while others criticise anthropomorphism based on religious beliefs that humans are fundamentally different from animals. However, Charles Darwin and Alfred Russel Wallace established with their theory of evolution that humans are indeed animals, and that the differences are in degrees and not of kind. This implies that an animal that can feel, for example, fear will not experience it exactly as humans do, but based on its species' emotional capabilities. One reason some people hesitate to say that fish feel fear when hiding from predators may be that we lack a specific term for ‘fish fear’. In contrast, we do have a precise term, ‘pectoral fins’, to describe the fish equivalent of ‘arms’. This lack of specific terminology can make it harder to acknowledge that the emotion of fear in fish might be just as different from human fear as pectoral fins are from human arms. Pectoral fins and human arms are, however, a case of homology, and have many of the same properties (Cass et al., 2021; Diogo et al., 2009). It can therefore be argued that not including some form of anthropomorphism as part of our toolbox to analyse animal behaviour and welfare leaves out an essential tool to understanding the behaviour and welfare of animals. Critical anthropomorphism was introduced as a means of distinguishing between trustworthy and naïve anthropomorphism. Three kinds of criteria for critical anthropomorphism can be found in the literature: (1) the creature claimed to hold the feeling is sufficiently close to us; (2) there are statistics that identify significant commonalities of test subjects' descriptions; and (3) the attribution of emotional states give successful predictions of animal behaviour (Karlsson, 2012).

## 4 MEASURING FISH WELFARE

As more or less explicitly stated in many of the frameworks and definitions of animal welfare described above, the fish's experience of the world is probably never going to be directly accessible. When one has a range of frameworks that define what welfare can mean for an animal, we need tools to identify welfare states and to draw a line between when welfare is good or when it is poor. Welfare indicators (WIs) are the tools that a stakeholder uses to evaluate the fulfilment of the welfare needs of various fish species and life stages, and are currently the foundations of modern assessment schemes for fish welfare (Browning, 2023; Stien et al., 2020b). Since the publication of the last briefing paper in 2006 (Huntingford et al., 2006) there have also been numerous advances in the development and application of welfare assessment models for fish, particularly in the field of aquaculture (e.g. Stien 2020; Folkedal et al., 2016; Pettersen et al., 2014; Stien et al., 2013; Tschirren et al., 2021) and some of them can be of use for developing similar assessments for fisheries. The next sections of this article will expand on some of them.

### 4.1 Welfare indicators in aquaculture

Ideally, WIs should provide adequate information regarding the degree that the identified needs are met or frustrated, and they can be classified in different ways. One way is to think of them as either input‐ or outcome‐based. Input‐based (indirect) WIs address the resources and environment the animals have and outcome‐based (direct) WIs address the animals themselves (e.g. Stien et al., 2020). For example, input‐based WIs such as water temperature and oxygen saturation give information about whether water quality is appropriate for the species and life stage in question, while an outcome‐based WI such as a high respiration rate can signify their effect on the animal. This example illustrates that input‐based and outcome‐based WIs are often paired and serve different purposes. Input‐based WIs are typically used to monitor the environment and make sure that conditions are as good as possible for the fish. It is, however, often very difficult to monitor all input parameters that can be of importance all the time and in all places. Outcome‐based WIs describing the fish and their behaviour are therefore necessary to evaluate the effects of these parameters directly on the animal. WIs can also be classified according to the discipline or setting they are applied and developed in. For example, farm‐friendly aquacultural WIs are often termed operational welfare indicators (OWIs). This classification includes all WIs that are practical and feasible to use on farms (see, e.g., Noble et al., 2018, 2020). More complex WIs that need additional processing and analysis after sample collection are termed laboratory‐based welfare indicators, see Noble et al. (2018). OWI tools and toolboxes can be refined further for specific aquaculture settings. For example, the LAKSVEL‐protocol for Atlantic salmon (Nilsson et al., 2022) refines earlier OWI toolboxes for Atlantic salmon and applies these toolboxes to a single specific rearing system (net pens) and life stage (sea phase). The protocol includes the input‐based OWIs oxygen, temperature and salinity and the outcome‐based group‐level indicators group behaviour, group appetite and mortality. In addition, the protocol also includes outcome‐based OWIs applicable at the individual level, including spinal deformity, emaciation, maturation, scale loss, skin bleeding, body wound, snout wound, jaw deformation, eye opacity, other eye injuries, opercula status, gill status and fin status. These individual OWIs were originally intended to be manually assessed by fish farmers. However, rapid development of automatic image analysis and artificial intelligence means that many of these measurements are now being included in camera systems, assessing thousands of fish 24/7. This constitutes a potential paradigm shift in the welfare monitoring of farmed fish and is addressed in the later section of this review on ‘Emerging Technologies for Observing OWIs’.

The development and application of WIs is not exclusive to the Atlantic salmon; WI toolboxes are also available for other aquaculture fish species such as European seabass (*Dicentrarchus labrax*), gilthead seabream (*Sparus aurata*), rainbow trout (*Oncorhyncus mykiss*), Atlantic lumpfish (*Cyclopterus lumpus*), ballan wrasse (*Labrus bergylta*), Nile tilapia (*Oreochromis niloticus*) and common carp (*Cyprinus carpio*) (Gutierrez Rabadan et al., 2021; Imsland et al., 2020; Noble et al., 2020, 2026; Pavlidis & Samaras, 2020; Pavlidis et al., 2024; Pedrazzani et al., 2020; Saraiva et al., 2022; Treasurer, 2018). These toolboxes are applicable in both commercial and research settings, and mostly have similar frameworks, in that they often consider both input‐ and outcome‐based WIs. However, as each species and life stage often have differing requirements for their welfare needs, thresholds for a specific WI must often be adapted to the species and life stage in question. This can also be applicable for fish with differing health statuses within the same species and life stage. For example, the welfare need to feed can be fulfilled by having the opportunity to successfully forage for an appropriate food item, but differing species and life stages have differing nutritional needs and also foraging behaviours, so any species or life‐stage‐specific toolbox should account for the diversity in foraging behaviours that the fish adopt to capture food. However, a key goal of each toolbox, as noted earlier in this article, is that it should include a sufficient range of WIs to address all known welfare needs of the fish.

### 4.2 Welfare indicators in fisheries

Welfare monitoring in fisheries is a less developed field of study, although recent research shows promising results. It is important to humanely kill the catch and not have fishes killed by the process of catching. It is also important to optimise by‐catch survival, either by avoiding the capture of by‐catch entirely or through the release of unwanted individuals alive once in the catch. There are examples of input‐based (e.g. temperature change, dissolved oxygen concentration and crowding) and outcome‐based (e.g. injuries, physiological metrics and vitality/survival) WIs being used experimentally in fisheries science (e.g. Anders et al., 2022; Humborstad et al., 2020; Marçalo et al., 2019; Veldhuizen et al., 2018), but these are not generally applied in wild‐capture scenarios to systematically assess catch welfare. One example of how relatively simple measurements can constitute important advancements in fish welfare assessment, for fisheries both recreational and commercial, are vitality assessments (Davis et al., 2021). Vitality assessments use a suite of reflex and behavioural responses to externally applied stimuli to infer the animal's ‘vital’ or survival status (Davis et al., 2021). Vitality is impaired when animals are in a state of physiological exhaustion, beyond which death may occur (Davis, 2010). Examples of vitality metrics include a righting response where a fish is turned upside down and given 3 s to right itself—failure to do so would indicate reflex impairment. Similarly, gently squeezing the tail of a fish should elicit an attempted escape response—failure to do so would indicate reflex impairment. These (and other) metrics have now been validated and are correlated with various physiological disturbances (e.g. exhaustion of tissue energy stores, oxygen debt) and are also predictive of mortality (Davis & Ottmar, 2006; Raby et al., 2012). Importantly, there are several reflexes that can be assessed, and some can be restored if fish are provided with appropriate recovery conditions (Lennox et al., 2024). Also relevant here is the simplicity of these indicators (these can be easily taught to fishers with no scientific training) and cost (they are free; Lennox et al., 2024). In addition, if fish being landed are repeatedly observed to be impaired, this provides the fisher with real‐time information that there is an opportunity to improve their handling practices. In short, the reflex impairment WIs empower fishers to make real‐time assessments of fish welfare and to adjust their behaviours accordingly, which is a game changer for fish welfare in this field (Lennox et al., 2024).

## 5 ONE HEALTH AND ONE WELFARE CONCEPTS

The One Welfare concept refers to the interconnectedness of human well‐being, animal welfare and environmental sustainability, and incorporates both the physical and social environment. This concept was first introduced as a vision of a framework to be developed with a commentary in the *Journal of the American Veterinary Medical Association* (Colonius & Earley, 2013), where they acknowledge the need to join the three disciplines related to welfare: human well‐being, social and animal welfare. One Welfare can be considered an extension of the One Health concept introduced in the early 2000s (WOAH, 2021) but with the additional focus on the mental states of the animals and a more holistic approach. The One Health framework also recognises the interdependence between human, animal and ecosystem health (WOAH, 2021). The five sections of the One Welfare Framework are (1) the connections between animal and human abuse and neglect, (2) the social implications of improved animal welfare, (3) animal health and welfare, human wellbeing, food security and sustainability, (4) sssisted interventions involving animals, humans and the environment, (5) sustainability: connections between biodiversity, the environment, animal welfare and human well‐being (Garcia Pinillos, 2018). All five sections are applicable to fish welfare.

There are many areas that would benefit from more studies within the One Health and One Welfare concepts; probably the most interesting area for fish welfare is ethics and social implications because of the controversy surrounding whether fish are sentient or not. Another area of interest relates the improvement of animal and/or farmer welfare to the improvement in productivity (mainly for terrestrial livestock; Orihuela, 2021) and fish (Kankainen et al., 2012). Studies on the One Welfare concept in fish show that a good and healthy physical environment leads to better fish welfare, both in the wild and under confined conditions (e.g. Prentice et al., 2025; Kleiber et al., 2023, and reviewed in Kristiansen et al., 2020). However, more studies relating social environment to fish welfare are needed, with results incorporated into sustainability indexes.

The COVID‐19 pandemic provided an opportunity to perform impact studies under the One Welfare concept (García Pinillos, 2021) through the combination of the zoonotic virus with its social and economic impact on the fish farming industry. A Scottish case study showed that the COVID‐19 pandemic affected all stakeholders involved in the fish farming production chain, from the fish farm to processing plants, including those involved in harvesting and fish transport, distribution, retail and ultimately consumers. It had a direct impact on the environment where both animals and workers interacted, but fish health and welfare were not compromised due to good coping responses from the aquaculture industry (Murray et al., 2021). However long‐term effects cannot be ruled out and it was obvious from the study that unexpected events like disease outbreaks could have put the system under greater duress.

## 6 CAPTIVE FISH WELFARE

### 6.1 Farming fish for food

**FIGURE 2:**
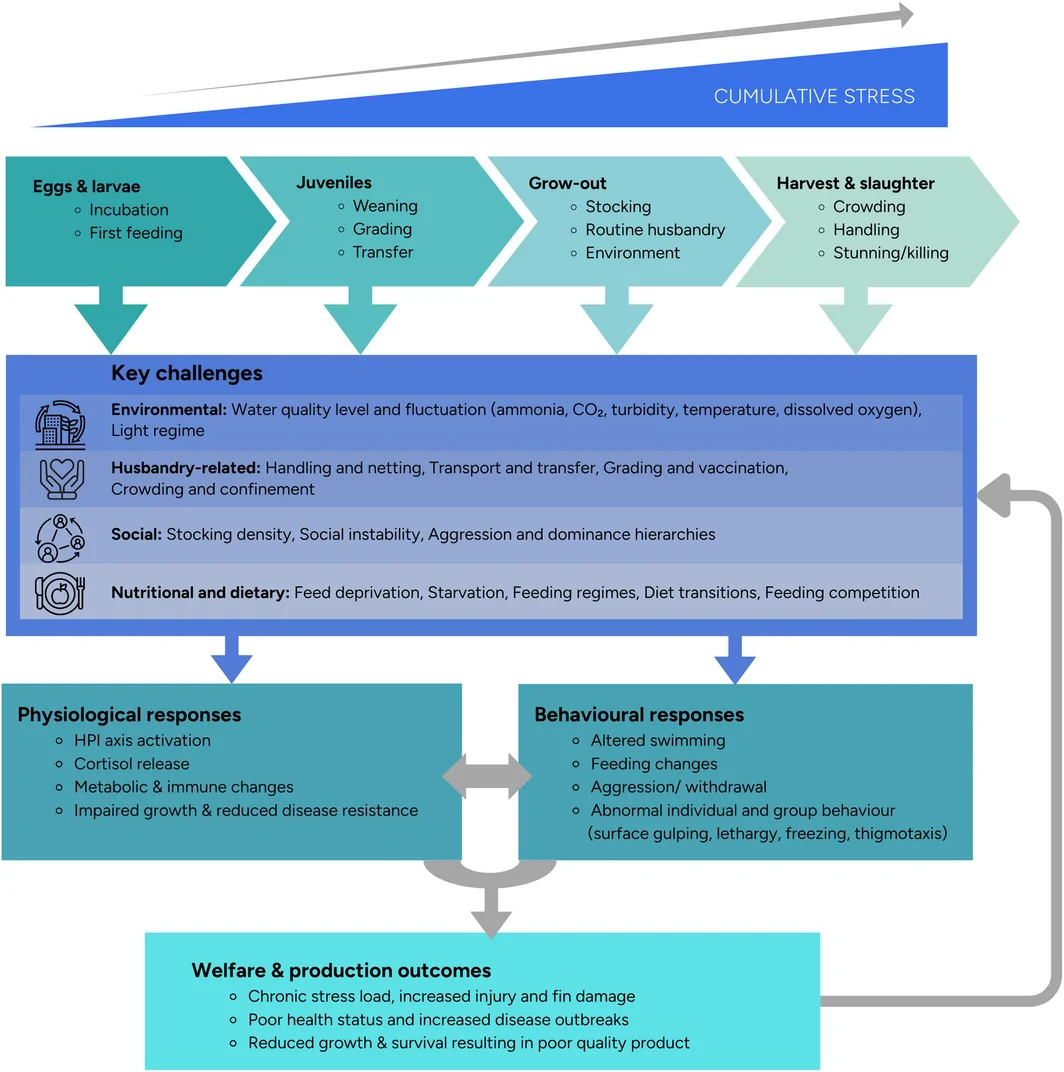
A simplified, integrated conceptual pathway illustrating the potential stressors that can be encountered by fish throughout the aquaculture production cycle, from early life stages to harvest, and their associated physiological and behavioural responses. Across production stages (eggs and larvae, juveniles, grow‐out and harvest), fish are exposed to overlapping potential environmental (e.g. temperature, dissolved oxygen, water quality, light regime), husbandry‐related (e.g. handling, grading, transport, crowding), and social and nutritional stressors (e.g. high stocking density, aggression, feed restriction, poor or inappropriate diets). These potential stressors converge on a central stress‐response cascade, initiating stress exposure and the activation of the hypothalamic–pituitary–interrenal (HPI) axis, leading to primary neuroendocrine responses, secondary metabolic, osmoregulatory and immune responses, and longer‐term tertiary effects on growth, health and resilience. Behavioural responses (including changes in activity, feeding, avoidance and abnormal behaviours) can interact bidirectionally with physiological stress responses. The integration of these processes results in downstream negative welfare outcomes (e.g. chronic stress, injury, poor health status) and production consequences (e.g. reduced growth, increased mortality and disease susceptibility). A similar figure could be used for challenges related to ornamental fish farming and fish used for research.

Aquatic animal production is expected to increase by 10% by 2032. Of the 223.2 million tonnes of aquatic animals produced in 2022, 49% (91.0 million tonnes) were from capture fisheries while 51% (94.4 million tonnes) were from aquaculture, surpassing for the first time capture fisheries in aquatic animal production. Asian countries (especially China) are the main aquaculture producers, focusing on mainly inland water production. Extensive and semi‐intensive aquaculture systems are increasingly being replaced by intensive aquaculture systems in many sectors (FAO, 2022). Intensification can cause potential fish welfare issues, for example due to increased stocking densities and disease outbreaks (unless the production systems are tightly controlled), and these challenges need to be considered during both the planning and operational phases of the farming operation (see Figure 2 for a simplified overview of main stressors, welfare consequences and welfare indicators for farmed fish). This is particularly important when new production systems are developed, a situation where we can learn from past experiences regarding their implementation (Vis & Europäische Zusammenarbeit auf dem Gebiet der Wissenschaftlichen und Technischen Forschung, 2013). Enhancing animal welfare often improves production and this can be a win‐win situation for farmers (Noble et al., 2012; Saraiva & Arechavala‐Lopez, 2019). In this section we address these potential issues and solutions in relation to the increased focus on fish farming worldwide, both concerning the fish themselves and the environment under the One Health and One Welfare concept.

Climate change is likely to further compound several stressors that are detrimental to fish welfare in both freshwater and marine aquaculture (Falconer et al., 2025; Woo & Subasinghe, 2023). For example, changes in temperature ranges and distribution, an increasing frequency of hypoxic conditions, freshwater restrictions that affect water quality, floodings and more frequent marine storms that in turn result in higher and longer wave periods, higher currents and wind speed have an important impact upon fish welfare (Cochrane et al., 2009; Tewabe, 2014). Other challenges that may appear include feed management under warmer temperatures (formulation, delivery, etc.), exposure to new harmful organisms such as predators or pathogens, or the increasing occurrence of biological catastrophic events such as jellyfish swarms or algal blooms (Woo and Subasinghe, 2023).

Climate change can also affect fish farmed for conservation purposes, where fish are farmed in captivity and released into the wild, impairing the balance between release survival and imprinting due to lower survival rates under those environmental challenging conditions (Braithwaite & Salvanes, 2010) that need to be solved at a species‐specific level. Imprinting is a specific type of long‐term memory related to early learning where young fish develop a preference for specific environmental (including olfactory or geomagnetic) or social cues (kin recognition) (Kimmel et al., 2023). If juveniles must be retained in captivity for extended periods to enhance post‐release survival, unintentional artificial selection can occur. This “domestication” process may reduce fitness and survival in the wild, thereby counteracting the intended benefits of prolonged rearing.

Domestication is the human‐induced selection process that gradually changes a cultured organism, extending over generations and involving developmental effects within each generation, culminating generally in genetic changes (Price, 2002). The effects of domestication and selective breeding on the welfare of farmed fish are far from being straightforward. When compared to farmed terrestrial animals, domestication and selective breeding of fishes is mostly very recent with the exception of, for example, common carp, where domestication may have begun around 3000 years ago, with the vast majority of farmed fish being under domestication and selective breeding merely since the middle of the twentieth century (Balon, 2004; Teletchea, 2015; Teletchea & Fontaine, 2012), while the domestication of mammals started over 10,000 years ago (Zeder & Hesse, 2000). Although considerable efforts have been undertaken to speed up the domestication process in fishes (Duarte et al., 2007), higher levels of domestication do not necessarily correspond to better welfare (Saraiva et al., 2018). One of the core issues is generation intervals, for example Atlantic salmon (*Salmo salar*), a key global farmed fish species, generally has a generation interval of 4 years (Gjedrem, 2000). Selective breeding programmes started in the 1970s (López et al., 2019), meaning there are ≥12 generations of domesticated salmon strains today (Glover et al., 2017). In addition, the maturity of artificial selection by the aquaculture industry is diverse across the >700 globally farmed fish species (e.g. Sonesson et al., 2023) and breeding programmes often focus on growth, flesh quality, nutritional quality and pathogen resistance (Sonesson et al., 2023). This targeted process may have unknown negative effects on welfare‐related traits through pleiotropic or epistatic mechanisms (Saraiva et al., 2018). What is known is that domesticated strains of farmed fish have higher standard metabolic rates and lower aerobic scope, lower resistance to hypoxia, inconsistent stress responses and can have higher aggression rates and risk‐prone behaviours (see Saraiva et al., 2018 for a review). These effects are also described for many other domesticated animal species (Jensen & Andersson, 2005).

### 6.2 Public aquariums, zoological collections and ornamental fish for home aquaria

There are still many challenges within the pet industry and the aquarium trade both for home and public aquariums and collections (Jones et al., 2022; Smith, 2023; Stevens et al., 2017; Walster, 2008). This industry is based on both wild‐caught fish and fish bred in captivity. More than 120 countries are involved (from Ornamental Fish International) in the ornamental fish trade, with more than 10,000 different fish species and billions of animals being traded annually (Torgersen, 2020).

Legislation that protects fish under farming or research also applies to the ornamental fish industry in some jurisdictions, but in many countries fish kept for ornamental purposes still lack legal protection. Fish are produced and internationally exported with no guarantees that the minimum fish welfare requirements are being addressed (Jones et al., 2022; Maia et al., 2025; Torgersen, 2020). The origin of traded fish is sometimes difficult to trace, and most fish are transported for long distances, often by plane and not always under optimal conditions. Traceability is a very important issue that should allow buyers to follow the health and welfare conditions of fish during their life cycle. Once pet owners take the fish to their homes there is often no follow up of the animals and no guarantee that their welfare requirements are being met, adding another layer of complexity to welfare issues.

To the authors' knowledge, vaccines are currently unavailable for ornamental fish, as most therapeutics are developed for farmed food‐fish species and are not tailored to the taxonomic diversity of the ornamental trade. Many medications can also be purchased over the counter or online without veterinary oversight. Additionally, frequent species misidentification can result in inappropriate husbandry, such as unsuitable water conditions, incompatible species combinations or excessive stocking densities, leading to increased aggression, chronic stress and compromised welfare (Maia et al., 2025; Sloman et al., 2011).

Stevens et al. (2017) suggested a series of interventions and research needed to improve the welfare of ornamental fish with respect to the multiple stressors they experience. Brandão et al. (2021) reviewed angelfish (*Pterophyllum scalare*) literature, one of the most common ornamental fish worldwide, and characterised different behaviours that could be related to their welfare state. Their review reflects on the importance of understanding the behaviour of the ornamental fish species to improve welfare.

Some OWIs have also been developed for ornamental fish (Jones et al., 2022) but there are no records or further publications outlining the use of these OWIs within the ornamental industry. Furthermore, species‐specific information and guidelines should be developed due to the high diversity of species being traded. The Ornamental Aquatic Trade Association (OATA; https://ornamentalfish.org/) contributes to improving this by raising awareness of fish welfare within the ornamental fish industry through publishing learning materials, biosecurity and health standards as well as adhering to the pet code of practice and the responsible pet ownership standards. OATA plays an active role in forming relevant standards to be applied to pet shop licensing and informing owners that there are certain legal responsibilities under animal welfare legislation. While owners who do not look after their fish properly could be committing an offence and face prosecution, there are few prosecutions regarding fish trade worldwide. OATA has not published any welfare indicator guides for producers or owners that are visible in their webpages. Most available books and papers are about caring for fish under research or farming conditions and very few publications regarding ornamental fish welfare are currently available (Jones et al., 2022). A specific study in Thailand, one of the major exporters of ornamental fish worldwide, on biosecurity and welfare that used OWIs, was rather discouraging as most pet fish stores were found to have poor husbandry in terms of fish health with 93% of fish having fin damage and 79% white spots on the skin (Department of Food Animal Clinic, Faculty of Veterinary Medicine, Chiang Mai University, Chiang Mai 50100, Saengsitthisak et al., 2020). The conclusions from their research indicated extremely poor animal welfare and poor husbandry in terms of fish health.

### 6.3 Fish used for scientific purposes

Fish have been widely used for scientific purposes in many ways, including those related to biomedicine, toxicology, physiology, behaviour, fisheries and aquaculture. Like other vertebrates, they are usually governed by national or federal legislation and overseen by an ethical committee for animal use (e.g. in North America they are called Institutional Animal Use and Care Committees). The ethical principles guiding research are generally based on the 3Rs: Replacement, Reduction and Refinement (Grunow & Strauch, 2023; Lauwereyns et al., 2024; Russell & Burch, 1959). In brief, ‘Replacement can be defined as methods, strategies or approaches that do not involve the use of live animals. Reduction covers any approach that will result in fewer animals being used to achieve the same objective. Refinement signifies the modification of any procedures or practices from the time the experimental animal is born until its death to minimise its suffering and enhance its well‐being […]’ (EU Commission, Animals in science; https://environment.ec.europa.eu/topics/chemicals/animals-science_en).

Fish as a group form a significant part of total animal experimentation in many countries. For example, in 2022, 14% of regulated procedures in the UK involved fish, the majority of which were zebrafish (UK government statistics of scientific procedures in living animals). At the European level (Eu‐27 and Norway), in 2022, fish accounted for over 30% of animals used for scientific purposes, with the majority being salmon, trout, char (*Salvelinus alpinus*) and graylings (*Thymallus arcticus*) (15.4%), followed by other fish species (8.2%) and zebrafish (4.3%). Animal experimentation committees assess whether the harm to animal welfare is justified by the goals (harm–benefit analysis). These deliberate manipulations under experimental conditions are generally based on the fundamental principles of harm–benefit analysis (HBA), with ‘harm’ defined as any negative impact on animal welfare addressed by the five freedoms (Brønstad, 2018; Brønstad et al., 2016). This analysis advocates that the expected benefits of a study should outweigh the harm imposed on animals in terms of knowledge gains (Würbel, 2017), which may include aiding the development of methods or products that could reduce animal suffering in the future (Brønstad et al., 2016). In the EU there is a ban (in some countries with the possibility for exceptions) on causing animals severe suffering during experimentation.

Severity assessments are key for implementing refinement goals when fish are used for scientific purposes (Hawkins et al., 2011), and are also addressed in many legislative frameworks, for example, Directive 2010/63/EU of the European Parliament and of the Council of 22 September 2010 on the protection of animals used for scientific purposes (European Union, 2010; http://data.europa.eu/eli/dir/2010/63/oj). Article 15 of the Directive states that all member states must ensure that all procedures are classified as either ‘non‐recovery, mild, moderate or severe’ as set out in Annex VIII of the Directive (European Union, 2010; http://data.europa.eu/eli/dir/2010/63/oj). To guide researchers with the classification Annex VIII also includes examples of procedures for each of the severity classes, and Hawkins et al. (2011) have provided guidance on applying these severity classifications for fish. However, severity assessment methods have sometimes been criticised for lacking rigour, transparency, consistency and fairness (Grimm et al., 2019).

Numerous articles have also addressed the welfare documentation of various fish species used for scientific purposes, including Atlantic salmon, rainbow trout, Atlantic lumpfish, ballan wrasse, European seabass, gilthead seabream, common carp, Nile tilapia, goldfish (*Carassius auratus*) and zebrafish (Toni et al., 2019; Golledge and Richardson 2024 in Chapter 48 on fish by Rey Planellas and de Leaniz; Noble et al., 2026). They outline the indicators to use in welfare documentation and various husbandry operations that a stakeholder should consider.

There are many ways refinement among the 3Rs can be considered. For example, when undertaking manipulations and surgeries that may negatively impact fish welfare, sedation or anaesthesia should be used to improve welfare outcomes (Hadfield & Clayton, 2021), unless it is deemed to have a greater detrimental impact on the fish than the procedure itself or is detrimental to scientific objectives (Schroeder et al., 2021). However, the use of sedatives and anaesthesia are themselves physiologically challenging for fish and, especially in field scenarios, may contribute to post‐release predation, emphasising the importance of developing context‐specific welfare maintenance plans (Cooke et al., 2016). Furthermore, fish kept in research facilities are often under controlled temperature and light conditions, where a caretaker can address their nutritional needs, and monitor their health and welfare. However, the fish can still experience welfare challenges as knowledge on their welfare needs may be lacking or incomplete, the amount of space they have may be limited, there could be infrastructure failures (e.g. problems with water supply) and they may be exposed to pathogens causing health problems or lack appropriate environmental enrichment.

Annex III of the directive (European Union, 2010; http://data.europa.eu/eli/dir/2010/63/oj) states that ‘Fish shall be provided with an appropriate environmental enrichment, such as hiding places or bottom substrate, unless behavioural traits suggest none is required’. Zebrafish is one example of where environmental enrichment is usually lacking as they are kept in well‐controlled, but barren tank racks (Stevens et al., 2021). Physical enrichment materials may limit the monitoring opportunities a researcher or caretaker has, as they can interfere with the observer watching the fish (e.g. refuges). However, recent reviews have addressed this topic in more detail (Gallas‐Lopes et al., 2023; Stevens et al., 2021). Some enrichment features can be introduced without interfering with fish observations, such as substrate, which is required to ensure good welfare for Mozambique (*Oreochromis mossambicus*) (Galhardo et al., 2011) and Nile tilapia (Mendonça et al., 2010). A blue background is also known to reduce stress in Nile tilapia (Maia & Volpato, 2013). Environmental enrichment can also promote natural behaviour, which in turn improves the quality and scientific relevance of the results. Enrichment and general husbandry plans could also reflect conditions used by fish in the wild (e.g. a fish that lives in crevices should be provided with structures that allow them to seek cover in that manner).

Fish under poor welfare conditions will generate unreliable or misleading data so keeping them under good welfare conditions is critical (Prescott & Lidster, 2017). However, when the aim of the experiment is to subject fish to a potential stressor, such as those that can be encountered in real world scenarios (e.g. under potential climate change scenarios or potentially stressful husbandry procedures), being able to document how these factors affect fish welfare is paramount for understanding their severity and fish welfare outcomes (e.g. European Commission, 2018).

Other things to consider include social isolation, which can affect fish behaviour and physiology (Galhardo & Oliveira, 2014; Shams et al., 2018) but which is sometimes used as a control in studies regarding fish social interactions (Brandão et al., 2015). In addition, tests about anxiety in fish models require subjecting the fish to an environment that makes them anxious (Maximino et al., 2010) while toxicological studies require constant interaction with toxic chemical compounds (Bojarski & Witeska, 2020). Other common short‐ or long‐term studies such as health challenges that inoculate the fish with virus or bacteria have high health and welfare impacts on the animals in provoking disease and sometimes even death, often without using anaesthetics or analgesics to mitigate the negative effects. However, as stated above, preventing unwarranted suffering is key to the use of animals for scientific purposes and is reflected in many national and international legislative frameworks. A way to address this is via the use of humane end points (Ellis & Katsiadaki, 2021) and WIs are often central to the development and application of these (Noble et al., 2026). Long‐term studies are also more likely to promote cumulative stress (Bateson, 2016) and can potentially harm the mental capabilities of fish (Sneddon & Brown, 2020), which are critical factors for fish under experimental conditions. Long‐term studies also occur in the field, often using various types of conventional and electronic tags (Matley et al., 2024). Once fish have recovered from the handling and tagging procedure their welfare should ideally not be impaired, although this is not the case for all tags and species (Macaulay et al., 2021; see also a later section on new technologies in this review).

## 7 WELFARE IN WILD‐CAPTURE FISHERIES

Capture fisheries span three primary sectors (as per United Nations Food and Drug Administration [UN FAO] categories)—commercial, subsistence and recreational. Here, we discuss each in turn with a focus on aspects unique to each sector. Welfare in wild‐capture fisheries differs from the welfare of farmed, pet or those used for scientific purposes in that (i) all welfare impacts tend to be more or less negative, that is, there is little scope to introduce positive welfare in some fisheries, and (ii) the duration of the interaction between a human and the fish is much more limited in capture fisheries. While captive fish are under human influence throughout their life, captured wild fish are retained for periods ranging from minutes to hours, or a few days at the most (unless the fish become subjects of capture‐based aquaculture or used for the ornamental trade). Although this limits the period of human–fish interactions, the impact may be more long‐lasting for individuals that are kept alive in live fish markets, that are released or that escape. Moreover, the anthropogenic impact of fishing activities also impacts the general welfare of fish through their life cycles.

### 7.1 Commercial fisheries

The scale of the welfare impacts from wild‐capture fisheries is enormous. Recent estimates suggest that the annual global catch of ~90 million tonnes kills between 1.1 and 2.2 trillion animals, which represents more than 90% of the total number of all food production animals excluding insects (Mood & Brooke, 2024). However, these estimates do not include animals caught and/or affected by illegal unreported and unregulated fishing, released unwanted catch (aka bycatch and discards), ghost fishing and habitat degradation. Furthermore, there is substantial scientific evidence demonstrating that commercial fishing practices can be severely stressful and injurious for animals in both retained and released catches (see reviews: Breen et al., 2020; Veldhuizen et al., 2018). Despite this scale and severity, animal welfare in commercial catches is under‐researched and rarely promoted within fisheries management (Kaiser & Huntingford, 2009; Metcalfe, 2009). There is, however, growing evidence that the introduction of welfare‐conscious fishing practices could lead to ethical, sustainability and product quality improvements in wild‐capture fisheries (Breen et al., 2020).

**FIGURE 3:**
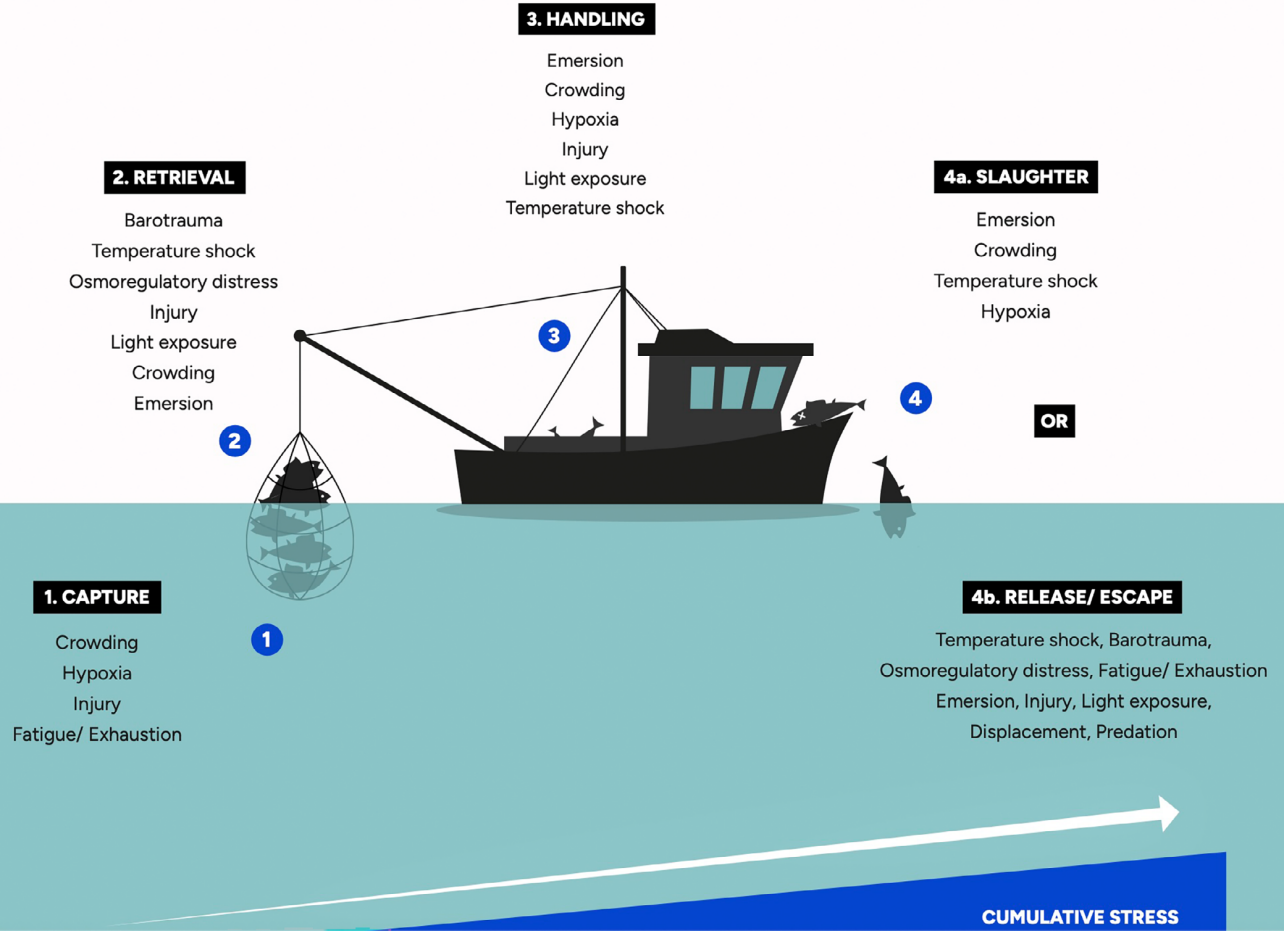
Conceptual pathway illustrating the potential stressors encountered by an animal during the capture process from the point of capture to its subsequent release or slaughter. ‘Retrieval’ is when the fishing gear and its catch are hauled from the fishing depth and brought aboard the vessel. ‘Handling’ is the process of removal of the catch from the gear, containment and sorting operations aboard the vessel. Adapted from Davis (2002), Broadhurst et al. (2006) and Breen and Catchpole (2021) by Boonstra for the Catch Welfare Platform.

It should be recognised that the introduction of good catch welfare practices to commercial wild‐capture fisheries is challenging. The capture process is a complex interaction between the capture technology, its operational environment, and the behaviour and physiology of the target animals (Breen et al., 2020). The FAO International Standard Statistical Classification of Fishing Gear recognises 58 different fishing methods in 11 broad categories (e.g. trawls, seines, gillnets, longlines, traps, pots) based on their construction and mode of operation (Nédélec & Prado, 1990). Each method has been developed to target species/taxa and operate under specific conditions, with respect to depth, hydrographic conditions and even season and time of day. This wide variety of capture methods and the environments in which they are used (in terms of latitude and hydrological, climatic and temporal regimes) means fishing operations can induce a wide array of different stressors on animals (Breen et al., 2020; Veldhuizen et al., 2018). The main stressors related to wild fisheries include exhaustion, injuries, thermal and osmoregulatory shock, barotrauma, asphyxiation, emersion and predation (Breen et al., 2020; Diggles et al., 2011; Veldhuizen et al., 2018; Waley et al., 2021) (see Figure 3). The response of individual animals also depends on many different factors, including species, ontogeny and the degree of exposure to those stressors (Sopinka et al., 2016). Furthermore, catches (including unwanted bycatch) comprise hundreds of different species from a wide range of taxa, all of which will have different welfare needs. Replacing or adapting current fishing methods with more welfare‐conscious practices is likely to initially impact productivity, profitability and employment, and therefore is likely to be resisted by the industry and other stakeholders (Browman et al., 2019). However, much can be learned about practical solutions to mitigate at least some of these stressors from capture‐based aquaculture (Humborstad et al., 2020). These solutions include limiting catch size to avoid crowding, injury and asphyxiation in the codend, for example, as well as reducing sorting and handling times at the surface; retaining the catch in water until they can be processed, thus avoiding emersion, exposure to UV light and extreme temperature changes, and careful and rapid handling while removing the animal from water and killing (or releasing) it (Breen et al., 2020; Humborstad et al., 2020; Veldhuizen et al., 2018). Beyond this, commercial wild‐capture fisheries remain a major component of global food security (Pinstrup‐Andersen et al., 2014), meaning banning, restricting or replacing common practices is likely to be politically challenging. Moreover, target species are often ‘non‐charismatic’ and their treatment during capture has historically held little interest with the public (Glas et al., 2019; Lundberg et al., 2019). It can therefore be seen that, even given moral and political will to introduce welfare‐conscious fishing practices, there are still issues of scale, complexity and practicality that could make them technically and economically prohibitive.

### 7.2 Subsistence fisheries

Subsistence fisheries are defined by the UN FAO as fisheries where the fish caught are consumed directly by the families of the fishers rather than being bought by middle‐(wo)men and sold at a larger market (FAO Fisheries Technical Paper 382–X2465). Subsistence fisheries are difficult to monitor but are exceedingly important to local communities and culture, particularly in rural areas. They provide a livelihood and food security safety net for some of the poorest people on the planet (Virdin et al., 2023). Many Indigenous rightsholders that fish do so as part of the subsistence fishing modality, although not all subsistence fishers are of Indigenous heritage and similarly not all Indigenous peoples or communities engage in subsistence fisheries. Indigenous fishers can operate in the commercial space (i.e. selling their catch), for ceremony (which is considered here under subsistence) or even in recreational space depending on the context of the activity. Fish welfare in the context of subsistence fisheries has been even less well studied than the commercial or recreational sectors. However, that does not mean that efforts to maintain fish welfare are not embraced by subsistence fishers. Subsistence fisheries often have a long history, knowledge and respect for fish (and other animals) that is passed down across generations and in some contexts codified in community cultural norms and laws. In many Indigenous communities, fish are ‘relations’ of the fishers hence ceremony and respect for fish is enshrined in how fisheries are carried out (Todd, 2018). Because most subsistence fisheries are inherently small scale, there is opportunity to devote significant time to the handling and euthanasia of individual fish. In subsistence fisheries, there tends to also be a culture of minimising waste such that bycatch is uncommon (i.e. most of the fish caught are harvested and that fishing ceases as soon as the necessary number of fish are captured). It is likely that there is much that could be learned from the lived experiences and knowledge of subsistence fishers to help refine practices for the other sectors.

### 7.3 Recreational fisheries

Recreational fishing is defined by the UN FAO as the ‘fishing of aquatic animals (mainly fish) that do not constitute the individual's primary resource to meet basic nutritional needs and are not generally sold or otherwise traded on export, domestic or black markets’ (FAO, 2012). Recreational fisheries occur in diverse habitats around the globe spanning freshwater and marine systems. Recreational fishing is the primary form of fishing activity in freshwaters of all industrialised nations and common in the coastal waters of wealthy nations (Arlinghaus et al., 2002, 2019). The scope and scale of the sector is massive, with more than 500 million recreational fishers around the globe. Although there are major deficiencies in monitoring of the sector, estimates of annual capture rates suggest that as many as 40 billion individual fish may be captured each year (Cooke & Cowx, 2004). Global estimates suggest that from total catches the overall catch and release rates may be on the order of 70% (Cooke & Cowx, 2004). Release rates of angled fish vary widely, with some species being almost always harvested (usually because of food value, e.g. walleye, mahi mahi) whereas for some other species nearly all individuals are released (e.g. muskellunge (*Esox muskenongy*), bonefish (*Albula vulpes*), Atlantic salmon), but these numbers vary a lot depending on the country. The reasons for releasing fish are complex and may be mandatory (e.g. to comply with harvest regulations such as bag limits, size limits, conservation or closed seasons) or can be voluntary. Voluntary reasons for release may be a result of angler conservation ethic (release a fish to grow, reproduce and potentially be caught another day), preference for specific fish for eating (either size or species), health concerns (e.g. parasite burden, contaminants) or their need for food (e.g. freezer is already full).

Recreational fishing can be conducted using a range of gears including some that almost always result in mortality (e.g. spear guns, rifles), but the most common type of recreational fishing involves hook, rod and line (known as angling). Across the sector there have been a variety of innovations intended to mitigate some of the welfare issues associated with recreational fishing. For fish that are to be harvested, there are now science‐based guidelines for humanely killing fish, which is good for fish welfare and for flesh quality, although it is unclear which of these methods have been embraced by anglers. One promising approach is the ike jime method (the use of a spike to rapidly destroy the brain) (see Diggles, 2016). Recent research in the aquaculture domain involving electroencephalogram (EEG) suggests that ike jime is effective when preceded by manual stunning (e.g. cerebral percussion; Gräns et al., 2025). However, the level of precision that is required is very high and varies among species such that phone apps have been developed to help anglers landmark sites for use of ike jime (Ike Jime App, available in the Apple Store). More research is needed for the development of effective killing methods (de Vis & Lambooij, 2016) that are effective and practical for anglers in the field.

Science has also revealed that some retention gear that is used prior to harvest (or for culling) can cause significant stress and injury (e.g. Madden et al., 2023) such that it is better to kill fish that will be harvested immediately. For fish that will be released, there have been hundreds of studies generating science‐based guidance intended to reduce injury, physiological stress, behavioural alterations and immediate and delayed mortality (reviewed extensively in Cooke and Suski, 2005; Arlinghaus et al., 2007; Brownscombe et al., 2017). What is apparent from that body of work is that there are many factors that are in the control of the angler (e.g. gear choice, angler behaviour) that influence outcomes. For example, choice of gears like barbless hooks that enable rapid release, minimising air exposure, and providing guidelines on environmental conditions which require special attention (e.g. barotrauma associated with fishing at depth, temperature, presence of predators) all influence fish welfare (Ferter et al., 2020). From a population biology and fisheries management perspective, mortality is the endpoint of concern. However, individuals matter in the context of welfare and in some cases individual impairments can scale up to influence populations. The key with fish welfare in the recreational fishing sector is to ensure that anglers are equipped with the best science‐based guidelines for interacting with fish (Brownscombe et al., 2017) and changing angling norms like minimising air exposure in fish, one of the major fish stressors, as has been clearly demonstrated by scientific research (Cook et al., 2015). Doing so can involve government regulations or, more commonly, voluntary actions inspired by various education and outreach activities, including sanctioning.

## 8 NEW DEVELOPMENTS AND TECHNOLOGIES

### 8.1 Emerging Technologies for Observing OWIs

As described in previous sections, a key aspect of applying WI toolboxes in the aquaculture industry involves manually inspecting fish welfare and health using well‐established individual‐based OWIs (Noble et al., 2018; Imsland et al., 2020; Pavlidis et al., 2024; Nilsson et al., 2022). Similar methods can also be used in research experiments conducted in tanks or pens (Toni et al., 2019; Noble et al., 2026) and for ornamental fish captured or bred for the pet market, as the fish are then equally available for direct inspection by humans. Although commercial and recreational fishing differ from these in that the window for human–fish interaction is shorter, the same principles are usually applied in that welfare assessment often involves inspection of individuals. When discussing fish welfare in a changing world, it is important to reflect upon how humans should monitor and document fish welfare given the prevailing changes and trends in this landscape. This is an area where emerging technologies can play an important role in enabling automatic evaluations of OWIs that may provide more accurate, precise and objective data. These solutions can also contribute to reducing the human labour requirements associated with welfare documentation, which in turn may minimise the need for direct interactions with the animal. In consequence, this may further improve fish welfare as welfare assessments will then be more consistent, repeatable and involve less handling.

The need for new indicators to measure the fulfilment of welfare needs has led to increased research and development efforts towards new fish monitoring technologies, a trend that is further stimulated by the recent developments within artificial intelligence (AI) and machine learning (ML). Today, a wide variety of different sensor systems and instruments are commercially available for monitoring fish welfare and the environment they are subjected to. These systems are mainly based on three different principles, which are optics (Saberioon et al., 2017), acoustics (D. Li et al., 2024) and biosensors (Hussey et al., 2015), all of which have their respective benefits and limitations. Many future solutions for monitoring OWIs are likely to feature the application of methods from ML/AI to acquire more insight from the collected data, irrespective of their origins (Fitzgerald et al., 2025).

The focus of the following section will be kept on outcome‐based OWIs. This is where the potential benefits of applying new technologies are highest; the drive towards improved measurements of input‐based indicators such as environmental conditions tends to be led by other sectors (e.g. agriculture) that measure the same indicators. This section distinguishes between approaches based on optics, acoustics and biosensors/telemetry and covers both individual indicators (i.e. describing welfare related parameters for individual fish) and group‐based observations (i.e. that provide information on fish groups).

#### 8.1.1 Optical methods

Most technological solutions aimed at fish welfare monitoring are based on optical measurements, usually featuring single‐ or stereo camera setups. Many of these methods target behavioural variables such as swimming speed and activity as they are often easier to assess optically than variables linked with physiology. For example, individual speeds have been measured with cameras (Pinkiewicz et al., 2011), a procedure that can be enhanced using AI methods (Li et al., 2021). While such individual observations may be linked with the welfare status of each individual fish, other approaches have aspired to measure group responses in fish such as feeding vs. non‐feeding behaviours or shoaling behaviour (e.g. Burke et al., 2024; Georgopoulou et al., 2024; Måløy et al., 2019). These responses can in turn be used to assess the welfare or stress status of the group under observation, exemplified by Eguiraun et al. (2018), who analysed video footage of a fish tank from above when the population was subjected to stressors (sounds generated by physically striking the external tank wall). The authors of that study used computer vision methods to derive the shoal centroid in the camera images and then calculated the Shannon entropy for the time series of centroid positions obtained when processing the full video recordings. The entropy of a variable describes its variability over time relative to its possible outcomes, and in this case, the entropy of the centroid was used to study how the shoal responded to the stressor. Other relevant examples can be found in Kumaran et al. (2025), who used cameras to monitor the spatial distribution of tank‐held Atlantic salmon in and around feeding periods by applying the ecological home range concept, and Ubina et al. (2021), who used an aerial drone to film the surface activity in a sea‐cage during feeding. The authors of the latter study also developed and trained a three‐dimensional (3D) convolutional neural network able to automatically quantify feeding activity, achieving a 95% accuracy in predicting manually determined feeding activity levels (labelled as ‘none’, ‘weak’, ‘medium’ or ‘strong’).

Although it is more challenging to assess the physiological and morphological condition of fish through optics, camera‐based methods are increasingly being used for automated disease and health monitoring in fish (Bohara et al., 2024). Specific examples of this include studies that use cameras combined with ML to efficiently detect externally visible wounds (Nissen et al., 2024) and signs of disease (Liu et al., 2023). There is also a similar trend towards this application area in the aquaculture industry, as evidenced by the current availability of commercial products offering automated lice counts and size estimation using AI methods applied to video (e.g. products from OptoScale AS, AquaByte AS, ScaleAQ AS among others). As implied in the previous section on OWIs, such systems are, besides providing these crucial production parameters, also excellent platforms for future automated assessment of several individual‐based OWIs such as wounds, deformities and individual movement speeds. For most such systems an expansion of application area would mostly require retraining the AI methods towards the detection of cues related to the desired OWI.

#### 8.1.2 Acoustic methods

While not as prevalent as optical solutions, acoustic tools have also been used to observe welfare‐related variables in both research and industry. Although some acoustic tools, such as split‐beam sonars, can be used to observe individual variables (e.g. swimming speed; Arrhenius et al., 2000), the main forte of echo sounders and sonars lies in their ability to observe fish distributions and groups. These observations can, when coupled with AI methods, provide useful outputs such as fish abundance, shoal shape and morphology (Baidai et al., 2020; David et al., 2024) or even detect behavioural changes due to disease outbreaks (Måløy, 2020). Recent studies have also implied the potential of combining AI with the high spatial resolution offered by multibeam acoustic devices in obtaining previously unobservable features such as the inter‐individual distance in commercial cages (Kristmundsson et al., 2023).

Passive acoustic monitoring (PAM) is another technology within the acoustic domain that has other advantages. When using PAM in the aquatic environment, hydrophones are deployed in an area and set up to monitor the aggregated sounds or ‘soundscape’ in that area. Unlike other acoustic methods that actively emit pulses to scan the spatial environment (e.g. echo sounders, sonars), PAM seeks to capture sounds produced by the environment and emitted by nearby organisms. This method is seeing increased use and has recently been applied in aquaculture research to observe group responses in fish when exposed to feeding (Helberg et al., 2024; Rosten et al., 2023) or stress (Jónsdóttir et al., 2024) in fish farms. When using high‐fidelity hydrophones, PAM will provide a soundscape that contains data on all acoustic features within a long distance from the hydrophone. However, the collected data usually spans a wide frequency range and high time resolution, leading to extensive and variable datasets that are difficult to process using conventional methods. This can be improved by the strategic application of AI‐based methods, rendering AI‐assisted PAM one of the more promising avenues for future innovations for non‐invasive assessment of fish welfare.

#### 8.1.3 Biosensors and telemetry

Biosensors and telemetry are methods where individual fish are equipped with embedded electronic devices that typically contain sensors measuring properties in or near the fish, and that either transmit the measured data (acoustically or through radio) or log them in an onboard storage medium for later retrieval (Thorstad et al., 2013). These devices are implanted into the body cavity of or attached externally onto the fish and are therefore considerably more invasive than optical and acoustic methods. Although this, together with the high cost per individual animal monitored, largely prohibits the use of this technology as a tool for continuous welfare assessment in the aquaculture industry. Biosensors are seeing increased use as a tool for welfare assessment within research (Brijs et al., 2021). However, when using these methods, care must be taken to ensure that the animals do not suffer adverse effects due to surgery, tagging or the presence of the tag (Macaulay et al., 2021; Matley et al., 2024; Wright et al., 2019), all of which may result in abnormal behavioural or physiological responses. Moreover, to ensure food security, all such devices used in aquaculture settings must be collected after production and prior to slaughter, which may be difficult and expensive to do. Despite these challenges, biosensors and tags have a major advantage in being in physical contact with the fish, unlike optical and acoustic methods, which rely on remote sensing. This proximity to the animal means that a vast new selection of variables, particularly linked with physiology, becomes possible to gauge (Brijs et al., 2021; Endo & Wu, 2019). In recent years, an increasing number of studies have used commercially available biologgers that measure heart rate in individual fish to evaluate welfare and stress (Hvas et al., 2020). Other studies have used accelerometers and/or gyroscopes to provide detailed information on fish locomotion (which is a manifestation of behaviour and physiology) and the energy expenditure of fishes (Cooke et al., 2016), the data from which have also been linked with stress in salmonids (Cooke et al., 2016). Data from such sensors are usually collected at sampling rates (10 Hz or more) that are much higher than those associated with fish movements and can thus capture most fish motions, such as tail beats and even feeding (Brownscombe et al., 2014). This has recently been exploited by using acceleration data to derive new data types such as relative thrust in locomotion (Warren‐Myers et al., 2023) or as proxies for respiration when used to sense opercular motions (Martos‐Sitcha et al., 2019). When using acoustic telemetry, it is also possible to get data on individual movements using the properties of the acoustic communication signal used to transmit the data. If an acoustic telemetry system features four synchronised receiver units, these can be set up to detect the 3D position through the time difference of arrival principle. Such data can in turn be used to estimate individual movements by finding the difference in consecutive 3D position samples. Preliminary studies have also verified the potential of applying Doppler shift calculations on the acoustic pulse emitted during transmission to thereby determine speeds directly (Hassan et al., 2022).

In addition to the more established principles described above, recent developments in miniaturisation and sensor technology have also enabled the direct measurement of new variables associated with fish physiology that were previously not possible to observe for living fish, including blood glucose levels (Wu et al., 2019), blood oxygen levels through photoplethysmography (Svendsen et al., 2023) and gastrointestinal blood flow (Brijs et al., 2019). While these and similar parameters are still at an experimental stage, their proof of concept shows that such variables could conceivably be available in future biosensor tools for fish monitoring. Moreover, the processing power and efficiency of modern tags or implants have now reached levels where simple ML algorithms are possible to run during operation without compromising battery life or other functionalities, potentially enabling the derivation of yet further OWI, for example by deriving cardiac output values based on photoplethysmography data.

### 8.2 Technologies that can promote welfare in aquaculture

#### 8.2.1 Improving fish welfare in tanks and pens

As the aquaculture industry has developed, an increasing number of new technological solutions that can promote fish welfare have become available. The most prominent example is the use of treatment procedures to combat pathogens and diseases or remove external parasites (Barrett et al., 2020). Most of these procedures were previously based on immersing the fish in medicinal chemicals or fresh water to kill the pathogens/parasites or stimulate them to release from the fish. However, in recent years, some of the organisms causing these challenges (parasites in particular) have developed a resistance towards the chemicals typically used, promoting the development and application of non‐medicinal methods (Barrett et al., 2020). These methods are often mechanical, entailing the direct removal of the sea‐lice, using brushes, high pressure water and/or water turbulence or thermal bathing the fish in low or high temperatures. To increase the effect the methods are often used in combination or in sequence, for example first freshwater and then thermal. Irrespective of which methods are used, the fish need to undergo crowding and pumping before treatment, since the fish are often transferred from their aquaculture production units for treatment. In sum this means that although such methods can remove or reduce the problems caused by the pathogens or parasites, they subject the fish to additional stress in the form of crowding and pumping, followed by the treatment itself. Such additional stress can in some cases prove more taxing for the welfare of the fish than the ailment they were originally intended to combat (Overton et al., 2019). An important pathway towards improved welfare in fish farming thus lies in the strategic development and application of new and more gentle technological methods to cope with parasites and other pathogens. Some recent developments are based on active removal of sea‐lice, such as lasers (e.g. Bui et al., 2020) or physically separating fish from parasites.

In the case of salmon lice, fish–parasite separation can be improved by covering the topmost section of the cage wall with impermeable tarpaulin to prevent the surface waters from entering the cage, and pumping water from deeper layers that are less likely to contain lice into the enclosed volume to ensure refreshment of water (Jónsdóttir et al., 2023; Nilsen et al., 2017). Submerging the entire cage to deeper layers is an alternative solution based on the same principles (Barrett et al., 2020), and that may be combined with shielded snorkels (i.e. tarpaulin covered tubes that extend from the submerged cage to the surface) that provide the fish with access to air (Oppedal et al., 2017). These approaches may provide first steps towards solving the lice issues in salmon farming, but they may also pose welfare challenges to the fish. For example, submerged cages without snorkels limit the fish accessing the water surface and if the species that is farmed needs this access to refill their swim bladder to aid their buoyancy (e.g. Atlantic salmon; Oppedal et al., 2017), additional measures must be put in place to allow for the fulfilment of this need (e.g. via the use of submerged air domes; Oppedal et al., 2020). If fish do not adapt to and sufficiently use the air domes, they can have problems with buoyancy, which can lead to various welfare problems, including vertebral deformities (Korsøen et al., 2009).

While closed production units (i.e. systems in which the production environment is separated from the ambient water by impermeable barriers and whose inlet water is controlled) and recirculation aquaculture systems (RAS) are primarily defined as production forms, they can also be considered technologies that can promote welfare in some instances (Kolarevic et al., 2014). Closed systems are usually equipped with filtered water intakes and sometimes use recirculated water, thereby limiting or even preventing fish from being exposed to pathogens and parasites. Moreover, being able to control environmental factors such as temperature, oxygen and water exchange could enable the farmer to actively avoid detrimental conditions and thus ensure acceptable welfare (Kolarevic et al., 2014; Timmerhaus et al., 2021). Controlling water exchange also provides a means by which to avoid other external threats that have negative impacts on welfare, such as jellyfish (Clinton et al., 2021) or toxic algae (Karlson et al., 2021). However, going from open to closed production systems is not without challenges, some of which could reduce fish welfare. The higher production costs in closed production may, for instance, mean that fish density must be kept higher than in sea cages to be economically viable (Dalsgaard et al., 2013). Furthermore, despite the higher ability to control water quality in such systems, suboptimal practices such as a water renewal rate that is too low can make closed systems more susceptible to low dissolved oxygen saturation levels or the buildup of substances such as carbon dioxide, nitrogenous compounds (Lindholm‐Lehto et al., 2025) or hydrogen sulphide (Letelier‐Gordo et al., 2020), which can lead to impaired welfare and mortality. Closed systems are also reliant on the continuous function of technologies such as pumps and oxygenation systems to sustain the production. Pump failure can, for example, lead to water circulatory failure, which will rapidly lead to suboptimal or even harmful production conditions if not restored. As for other production systems, potential welfare challenges of closed systems also include reduced opportunities to exercise if current speeds are too low and anthropogenic noise at the facility (Radford & Slater, 2019). Most tank production systems, including RAS, are kept as barren environments that could lead to abnormal or aberrant behaviours due to lack of stimulation.

While some welfare‐promoting technologies arise due to industrial needs, other solutions may emerge from research. The economic incentive to develop such solutions is thus not as strong as in the case of reducing the impact of stressors, but the emergence and application of such solutions may in the end prove equally important as generally improved welfare conditions will lead to robust fish that are more resilient to external stressors than fish subjected to chronic stress due to suboptimal conditions.

Technological advances in the provision of environmental enrichment include water currents that promote fish exercise in addition to improving water renewal and quality (Villalba et al., 2024), and air‐bubble curtains, a physical and sensory enrichment that improves cognitive performance, feeding predictability and the overall health of the fish (Amichaud et al., 2024). Studies on sensorial stimulation by lighting (intensity, frequency and position) have also reported direct induced effects on behaviour such as altered vertical positioning and sustained swimming, while enhancing growth and reducing stress (Bui et al., 2013; Herbert et al., 2011; Wright et al., 2015). Therefore, while natural environments cannot be fully recreated in farming systems, the objective when designing enrichment can be to modify elements of the artificial environments to provide some of the stimuli of the natural environment and thereby create welfare benefits without compromising the biosecurity of the farms (Arechavala‐Lopez et al., 2022), and technological innovations will help advance this field.

#### 8.2.2 Precision farming and digital twins

Precision aquaculture, that is, the improvement of aquaculture practices through targeted application of technology and automation principles, can also be seen as a future technology that can promote welfare albeit in a less direct manner than the solutions described above (Føre et al., 2018). One of the main avenues for realising precision fish farming is the development of digital twin technology for aquaculture, wherein sensors are combined with mathematical models to achieve a more detailed and continuous overview of the various aspects in the aquaculture production process (Føre et al., 2024). The main idea behind digital twins is that they should represent all important dynamics in the target system or process, and in the case of fish in farms, this means all aspects of the fish's biology (i.e. behaviour, physiology/growth and welfare) in an aquaculture setting. Potential applications of digital twin technology in aquaculture include providing informed decision support during production and operations, serving as a virtual arena for personnel training, predicting future states or ultimately full feedback‐controlled production. Future digital twins of fish farm biology will thus need to combine sensor technology for observing WIs with mathematical models able to predict future welfare states in the fish. At present, there exist no models able to make such predictions, but future refinement of existing models of fish biology can provide a foundation for the development of such models since welfare can often be deduced from behavioural changes and physiological states (Giske et al., 2025). However, the potential welfare and health factors that should be considered in digital twins are addressed in a number of recent articles (Budaev et al., 2024, 2025; Giske et al., 2025; Noble et al., in press).

### 8.3 Technologies that can promote wild animal welfare in capture fisheries

The development and implementation of good welfare practices for different fisheries will require a thorough, research‐based assessment of the severity and timing of stressors, including their impacts on wild animals. To achieve this, it will be necessary to develop technological solutions for monitoring stressors and appropriate scientific methods for determining welfare, analogous to OWIs used in aquaculture (e.g. Noble et al., 2018; Pavlidis et al., 2024). This knowledge and toolkit could then be used to identify how and when in the capture process mitigating measures could be applied to improve welfare most effectively.

Ensuring acceptable welfare is more challenging in capture fisheries than in aquaculture because the interaction between humans and fish is limited and usually occurs only as the catch is brought aboard the fishing vessel to be sorted and processed. However, OWIs combined with improved fishing gear selectivity and marine area management could be used to minimise unwanted catch, which inherently improves the welfare of any potential bycatch (Breen et al., 2020). The continued research and implementation of technical measures to reduce unwanted catches will significantly improve the welfare of the unwanted components of the catch in some fisheries (ICES, 2023). Such innovations include traditional mesh size increases (e.g. Arkhipkin et al., 2023) and insertion of technical devices (e.g. Eigaard et al., 2021) to facilitate the escape of unwanted bycatch. More high‐tech solutions include video and broadband acoustic surveillance to determine the size and species composition of target schools before setting the fishing gear (e.g. Trawleye, CatchCam, DeepVision; Birch et al., 2022; Krag & Savina, 2022). However, the development of AI technologies to drive species recognition linked with active selection mechanisms could revolutionise fishing gear selectivity and the welfare of unwanted catch in the future (e.g. Smart trawl and Game of Trawls; Abangan et al., 2023).

Such innovations may also have synergistic benefits for the retained catch (Kennelly & Broadhurst, 2021). However, it may be necessary to prioritise the welfare of discarded or escaped individuals over the retained catch because these animals may live with reduced welfare much longer than fish that are slaughtered. Furthermore, the long‐term survival of released animals is likely to be beneficial for the sustainability of the fishery.

Releasing unwanted catch early in the capture process, before it is brought to the surface, has clear welfare benefits, that is, avoiding all the stressors associated with the ascent to the surface as well as exposure to air and handling aboard the vessel (see Figure 1). However, escaping through the selective mechanisms described above can still be injurious and stressful for fish, and has been shown to induce significant mortality, particularly in smaller individuals (Ingólfsson et al., 2007; Sangster et al., 1996; Suuronen, 2005). Protecting the catch in the codend (i.e. the net bag at the end of a trawl where the catch collects) from injury, crowding and asphyxiation can substantially improve survival in these escaping fish (Breen et al., 2007), as well as the quality of the landed fish (Brinkhof et al., 2018). To this end, commercially available protective codend systems are being developed (e.g. the Modular Harvesting System aka FloMo; Moran et al., 2023) that do not detrimentally affect the selective properties of the codend (Millar et al., 2023).

Physical impact from towed fishing gear on the seabed, and its contribution to habitat degradation, could be reduced with several technological innovations. Trawls can be made lighter to limit their drag and penetration into the seabed (e.g. Guijarro et al., 2017). Instrumentation to measure impacts and vibration can allow fine tuning of warp length and vessel speed to effectively lessen drag, crushing and sediment resuspension (e.g. Sala et al., 2009, 2019). Alternatively, the doors can be ‘flown’ above the seabed in a semi‐pelagic mode, thus reducing the overall footprint of the trawl track (e.g. Eighani et al., 2023). Similar hydrodynamic principles can be used to lighten trawl ground gears (Larsen et al., 2018), as well as dredges (Shephard et al., 2009) and beam‐trawls (Burgaard et al., 2023). Furthermore, these systems are also likely to contribute to significant reductions in drag and fuel usage that, in combination with other energy saving measures (e.g. Chen et al., 2023; Ng et al., 2020; Notti et al., 2016), will also help reduce commercial fishing's contribution to greenhouse gas emissions. Electrification of beam trawls (e.g. the pulse trawl) has shown that electrical stimulation of benthic fish and shrimp ahead of the trawl mouth reduces the need for heavy chains and consequential physical impact on the seabed (e.g. Depestele et al., 2019; van Marlen et al., 2014; van Overzee et al., 2023; Verschueren et al., 2019). Although the International Council for the Exploration of the Sea (ICES) advise that ‘there are fewer ecological and environmental effects of using pulse trawls than traditional beam trawls’ (ICES, 2018), concerns have been raised about the potential impact of this technology on the welfare of the targeted and untargeted catch, such as mortality and spinal injuries (e.g. de Haan et al., 2016; Desender et al., 2017; van der Reijden et al., 2017). Finally, protection of particularly sensitive habitats greatly benefits from technologies and programmes that enable accurate descriptions of their community structure and locations (e.g. Krag & Savina, 2022), along with effective management of fishing activities in such areas (e.g. De Juan & Lleonart, 2010; FAO, 2024).

Static gears also contribute to habitat degradation as marine litter from abandoned, lost or discarded fishing gear (ALDFG), in addition to causing collateral mortality through ‘ghost fishing’ (Gilman, 2015; Gilman et al., 2016; Macfadyen et al., 2009). There are several technological solutions for mitigating ALDFG in addition to programmes for reporting and recovery of lost gear, as well as facilitating onshore gear disposal to avoid abandonment at sea (Gilman, 2015; Gilman et al., 2016; Macfadyen et al., 2009; Mengo et al., 2023). For example, high‐tech solutions include relocation systems which broadcast the location of lost gears (e.g. PingMe and Ghost Fishing Solutions) and systems that use delayed deployment of recovery lines to the surface to avoid entanglement with marine mammals, etc. and enable recovery of lost gears (e.g. ResqUnit). Low‐tech solutions also exist to release unintended catch through escape openings and biodegradable components to render lost gears ineffective over longer periods of time (e.g. Dsolve and Innovative Fishing Gear for Oceans [INdIGO]; Brakstad et al., 2022; Cerbule, Grimaldo, et al., 2022; Cerbule, Herrmann, et al., 2022; Grimaldo et al., 2019, 2020; Grimaldo, Herrmann, Tveit, et al., 2018; Grimaldo, Herrmann, Vollstad, et al., 2018). Furthermore, the Marine Stewardship Council has recently revised its assessment criteria and standards to encourage the adoption of such practices and technologies (McLennan et al., 2023). One example of a relatively simple change that could potentially make substantial improvements to aquatic animal welfare is an initiative by the Norwegian government to both improve catch welfare and reduce the risk of ghost fishing. In 2020, following several cases of fishing gear left at sea for months, the Norwegian Harvesting Regulations were amended in 2025 to mandate that all commercially and recreationally operated gill‐nets, longlines and traps must be retrieved every 1 to 7 days, depending on the target species.

The stressors associated with retrieval, surface handling and slaughter (Figure 3) are thought to be particularly detrimental to the welfare of the catch (Breen et al., 2020; Veldhuizen et al., 2018). However, catch limitation systems for commercial trawls have been successfully developed and tested (e.g. Grimaldo et al., 2014), even in industrial scale pelagic trawls (e.g. Ingólfsson et al., 2022). Furthermore, fishing vessels are now being designed and constructed to incorporate welfare‐conscious handling of the catch (e.g. EcoFive), with the specific aim of improving catch quality.

Finally, in recent years, there has been an emergence of electrical immobilisation/stunning systems and procedures specifically for use in commercial fishing operations which advocates are promoting as ‘humane stunning/slaughter methods. However, there is currently no scientific proof that these systems are effectively rendering the fish unconscious as opposed to just immobilising them (tonic immobilisation) and further research is needed to test these methods.

In recreational fisheries, gear innovations have included new hook designs that are less likely to cause injury or mortality (e.g. circle hooks) and net materials that reduce physical injury (reviewed in Brownscombe et al., 2017). There have also been efforts to develop various topical agents that can be applied to injuries (e.g. sprays) that purport to stop bleeding or expedite healing but given that the industry producing such agents is unregulated, those claims have not been substantiated. There have also been innovations in gear used for handling fish, including devices that are supposed to improve hook removal, but again those claims have rarely been tested and when they have there has been some evidence that they do more harm than good (Cooke et al., 2021). Most of the innovations in the recreational fishing space with respect to gear are not about the gear itself but how anglers interact with the fish. Organisations like KeepFishWet.Org have been pushing for simple science‐based actions that benefit fish that will be released, including minimising air exposure and handling and reducing interaction with dry surfaces (Danylchuk et al., 2018).

## 9 SUSTAINABILITY AND FISH WELFARE

### 9.1 The sustainability goals and fish welfare

The officially endorsed worldwide definition of sustainable development is the one in the UN report Our Common Future (Secretary‐General & Development, 1987): ‘Sustainable development is development that meets the needs of the present without compromising the ability of future generations to meet their own needs’. The report called for worldwide sustainable development. The focus of the sustainability goals is largely on the needs of humans rather than animals and their welfare. As research and regulations are generally made under either sustainability or welfare perspectives, the resulting policies are often contradictory. This becomes exacerbated by the fact that, in aquaculture and fisheries, animal welfare science is much more recent than sustainability approaches. It is therefore essential to acknowledge that environmental protection and animal welfare, albeit close, are essentially different things and may even be antagonistic in some circumstances. For example, selecting for production efficiency can trade‐off against welfare, feeds with reduced fish content can be challenging for carnivorous farmed fishes, environmental regulations may prohibit the location of fish farms in more welfare‐suitable sites, land‐based intensive systems may bring environmental benefits but may also create welfare challenges, and disease and parasite control can trade‐off against welfare. All these examples are detailed in the present text and identifying conflicts between desired environmental and welfare outcomes as early as possible will allow for knowledge‐based consideration of trade‐offs using the best available evidence (Macaulay et al., 2022).

### 9.2 Blue foods

It is known that the environmental footprints of aquatic food plants and animals (blue foods) are lower than those of land‐based foods. Blue food systems can play essential roles in achieving sustainable development goals (SDGs) (United Nations, 2015) such as eliminating hunger and improving health, increasing the sustainability of oceans and inland waters, human rights, environment, equity and equality (Assessment, 2021). In the view of the FAO (Blue Transformation – Roadmap 2022–2030, 2022) the growing demand for blue foods requires a sustainable transformation, the Blue Transformation (BT), while supporting the SDGs. For the BT, the FAO selected three guiding SDGs (no poverty, zero hunger and reduced inequalities) to maximise the contribution of aquatic food systems to food security and the improvement of nutrition in a sustainable way.

This shows how sustainability has evolved and has been operationalised over the past three decades. It shows that the welfare of food animals has not been included in influential sustainability frameworks such as the SDGs, even though some researchers have argued in favour of a ‘mutually beneficial relationship between improving animal welfare and achieving SDGs’ (Keeling et al., 2019). This lack of focus on animal welfare within discussions of sustainability is remarkable given that, for instance, a reduction in stress in farmed fish contributes to improving feed conversion (see, e.g., Arechavala‐Lopez et al., 2022). An improved feed conversion leads to a reduction in the ecological impact of the aquaculture sector and to lower costs. Feed and diet optimisation can also play a major role in improving the sustainability of aquaculture production, especially in highly produced species (see Teodósio et al., 2020). However, fish nutrition research has often had a major focus on reducing the capture of fish as raw material for fish feed and performance metrics such as growth. In addition, increased reliance on farmed fish species as blue foods can increase food‐feed competition (the allocation of resources that can be used to directly feed humans to make animal feed instead, a current but unsustainable practise not well documented for aquaculture, see van Riel et al., 2023). Ingredient choice is paramount to reduce food‐feed competition, yet welfare must also be considered when balancing feed formulation regarding palatability, attractability and nutritional aspects. This relationship between welfare, feed production and sustainability is therefore complex.

In commercial fisheries, when damage to and stress in captured fish are reduced, the survival rate of the captured fish that are released into the water and of fish that are allowed to escape from the fishing gear due to the presence of a selectivity device will increase. The anticipated reduction in stress and damage will contribute significantly to more sustainable fisheries (Veldhuizen, 2017; Veldhuizen et al., 2018).

### 9.3 Resilience as a strategy to improve sustainability in aquaculture

The adaptive process for actively maintaining stability through change in response to various challenges is called allostasis (Sterling, 2012), which is crucial for good health and animal welfare (Korte et al., 2007). At the level of the individual, resilience has been defined as the ability of an animal to adapt to stressors and its ability to return to the ‘normal’ or previous state (Urruty et al., 2016), whereas robustness is the ability to maintain desired levels of agricultural outputs (for fish, aquacultural outputs) despite the occurrence of disturbances (see, e.g., Ten Napel et al., 2011). The resilience of fish implies, therefore, that the animal can reach allostasis.

A combination of housing conditions and events can lead to allostatic overload in fish due to cumulative effects, that is, the animal is overtaxed due to stressors (Arechavala‐Lopez et al., 2022), resulting in chronic stress‐related physiology and behaviour, making the fish more susceptible to disease (Yada & Tort, 2016). This increased susceptibility to diseases represents a major challenge as most species are farmed in open systems during the on‐growing phase. In these open systems, fish are exposed to pathogens and other harmful organisms (Van De Vis et al., 2020). Therefore, to make the production of food fish more sustainable, a structured approach is needed. In aquaculture, a strategy to improve the resilience of fish implies that steps must be taken by farmers to counter or mitigate the adverse effects of, for instance, climate change or other unpredictable events (Abisha et al., 2022). To improve the resilience of fish in aquaculture, four strategies are available in a similar way to those for terrestrial livestock production (Rebel, 2022): breeding programmes, nutrition, the use of probiotics and or immunostimulants, and enrichment. These four strategies are briefly discussed below.

#### 9.3.1 Breeding programmes and genetically modified fish

The focus in breeding programmes is the selection of genetically improved animals for the next generation. After reproduction, a genetic evaluation of offspring is performed to select offspring with the best breeding characteristics. These fish will serve as parents for the next generation (Blonk, 2010). However, breeding programmes used for fish do not necessarily correspond to better welfare because selection can be focused on optimising growth, although for numerous species there is a focus on pathogen resistance (see, e.g., Sonesson et al., 2023). A focus on growth may, however, lead to negative effects on welfare‐related traits (Saraiva et al., 2018). For improvement of resilience, an increased number of breeding programmes must be focused on traits like disease resistance or desired behavioural traits (e.g. less aggressive). Additionally, individual phenotypic differences in behaviour or coping styles/personalities within individuals and between species will influence the resilience to different environments and the domestication process (Castanheira et al., 2017).

Genetically modified (GM) fish have been used for research purposes for several decades as experimental models across a wide range of disciplines, most notably in zebrafish and medaka (*Oryzias latipes*) (see Muir, 2004). More recently, research has also focused on applying GM techniques to food production species, for example Atlantic salmon, although other species are increasingly being explored (see recent reviews on GM fish for farming in Wang et al., 2021; Robinson et al., 2024). Although controversial, some countries have approved the commercial use of GM fish. Consequently, the potential welfare implications of such genetic modifications, as well as the environmental risks associated with the escape of GM fish into wild populations, require careful assessment.

#### 9.3.2 Development of the immune system by vaccination and pro/pre‐biotics

Vaccination is widely applied in aquaculture to stimulate the immune system of fish, thereby enhancing their resilience to disease and reducing the need for therapeutic treatments during outbreaks of pathogenic microorganisms. In addition to vaccination, prophylactic strategies such as the use of probiotics and prebiotics have been increasingly explored and show promise in mitigating the negative impacts of pathogen outbreaks (Torres‐Maravilla et al., 2024). Together, these approaches support immune development and contribute to improved resilience of fish in aquaculture systems.

#### 9.3.3 Enrichment

Environmental enrichment is a major measure that is linked directly to fish welfare and health, thereby improving the resilience of the animal (see e.g. Manuel et al., 2015). By environmental enrichment, the complexity of the rearing environment is enhanced. This is necessary to meet the biological needs of the animals (Arechavala‐Lopez et al., 2022) and this is addressed widely in earlier sections of this article.

Enhancing the resilience of fish is a challenge due to the biological differences between farmed fish species and the variety of rearing systems and husbandry operations used worldwide. Biological differences between life stages within a given fish species must also be considered. Enhancing resilience raises the question of whether we should operationalise it for the sole purpose that fish can cope with suboptimal conditions. The premise of implementing measures to increase the resilience of a fish species ought to be that the animal can flourish during rearing.

### 9.4 Sustainability in fisheries and fish welfare

The stress experienced during wild capture can be severe enough to induce significant mortality among animals encountering fishing gear from several, often unaccounted, sources. This includes unwanted components of catch that are discarded and slipped (Breen & Catchpole, 2021; Huse & Vold, 2010; Tenningen et al., 2012), ghost fishing (Brown & Macfadyen, 2007; Macfadyen et al., 2009), selective escapees (Breen et al., 2007; Ingólfsson et al., 2007; Suuronen, 2005) and collateral impacts on benthic habitats and communities (Buhl‐Mortensen et al., 2014; Eigaard et al., 2016; McLaverty et al., 2024; Shephard et al., 2013). Such unaccounted mortality threatens the sustainable management of marine resources (Breen & Cook, 2002; Chopin et al., 1996; Gilman et al., 2013).

Stress immediately prior to slaughter has been demonstrated to reduce flesh quality (Poli et al., 2005), including in several commercially caught species: Atlantic cod (*Gadus morhua*) (Digre et al., 2010; Kristoffersen et al., 2006; Olsen et al., 2008; Stien et al., 2005), Atlantic herring (*Clupea harengus*) (Roth & Skåra, 2021), Atlantic mackerel (*Scomber scombrus*) (Anders et al., 2020, 2022), haddock (*Melanogrammus aeglefinus*) (Digre et al., 2010; Karlsson‐Drangsholt et al., 2018) and turbot (*Scophthalmus maximus*) (Morzel et al., 2003). While quality is not always directly correlated with landing price (Sogn‐Grundvåg et al., 2021), less stressful capture methods (e.g. longline) generally have a good reputation for quality and thus attract better prices at auction (Sogn‐Grundvåg et al., 2020). Furthermore, poor quality can limit value‐adding opportunities further down the supply chain (Sogn‐Grundvåg et al., 2022), as well as detrimentally affect storage and shelf life of fisheries products (Anders et al., 2022), thereby increasing wastage and further impacting sustainability. Therefore, the introduction of welfare‐conscious fishing practices that minimise stress has the potential to improve both the sustainability and quality of the landed catch.

It is important not to overlook the fact that sustainability is multifaceted, including economic and social elements, in addition to the commonly perceived conservation of the environment and harvested stock. Wild‐capture fisheries are also vital for global food security, particularly for developing nations. Seafood provides a substantial part of daily animal protein and micronutrient intake for more than 3 billion people (Troell et al., 2019). Also, more than 120 million people in the world depend directly on fisheries‐related activities (fishing, processing, trading) for their livelihoods, with most of these living in developing or emerging countries (HLPE, 2014). Therefore, to achieve truly sustainable fisheries, it is essential to consider the trade‐offs and synergies between these different elements of sustainability.

Finally, it is not only the fishing industry that should take responsibility for the welfare and sustainable use of the catch it handles. The scientific community catch and kill many hundreds of thousands of fish each year as part of regular stock assessment surveys to promote the sustainable management of fisheries. Recent discussions in ICES raised concerns that survey design and handling practices could be improved with regards to catch welfare and sustainability, particularly in terms of the number of animals caught relative to the required sample size, as well as effective handling and killing practices. In response, it has been proposed that ICES establishes an expert group that will, among other goals, recommend welfare‐conscious practices for ICES coordinated scientific surveys.

## 10 SOCIETAL RECOGNITION

### 10.1 How is fish welfare perceived by the public?

Despite the key role of fish in many aspects of human life, fish welfare has not been a top priority in public debates on animal welfare in many countries. Studies indicate that fish rank low on the socio‐zoological scale proposed by Arluke and Sanders (1996). This scale, which develops over time and varies across cultures, rates animals as more or less important to people, and therefore more or less worth protecting, according to several factors. These include how useful the animals are, how closely one collaborates with the individual animal, how cute and cuddly the animals are, how harmful the animals can be and how ‘demonic’ they are perceived to be. Since humans typically do not interact directly with fish and they come from an environment that is very different from ours, it may be expected that they rank lower, and that people are less able to feel empathy with them than with, for example, many of the mammals and birds they encounter. This is demonstrated in three European studies conducted between 2005 and 2010. In a study of Dutch consumers' views on farmed fish and pig production, Frewer et al. (2005) found that for a majority of respondents welfare‐oriented production practices were less important for fish than for pigs. However, the differences were rather small. This contrasts with a study of views among the Finnish population where only just over half of those who thought that chicken could feel stress also thought that salmon can feel stress (Kupsala et al., 2013). Furthermore, in a survey of the Norwegian public, Ellingsen et al. (2015) found that fish ranked lower than dogs, cows and chicken when it comes to ‘the right not to suffer’. However, as with the Dutch study, the difference was not big. Some recent European surveys that have not been published in peer‐reviewed articles point in the same direction and indicate that even though people, at least in Europe, may care less about the welfare of farmed fish than about the welfare of other farm animals, they still care quite a lot (Simcikas, 2020). However, a European survey reporting data from nine different countries and >9000 participants has also reported that 91% of participants believed fish welfare should be protected to the same/greater extent as for other animals that humans eat (https://www.ciwf.eu/media/7458798/2024-eu-aquatic-animals-survey_ciwf_efa_sapience_results-slides_v3.pdf). It may therefore be expected that for many people fish welfare is becoming more of an issue than it has hitherto been.

Although fish welfare perception seems not to be a global concern, there are studies indicating a sizable willingness to pay for farmed fish produced with extra focus on animal welfare by different groups of consumers. One study found that Danish ‘consumers were generally willing to pay more in premium for fish that had a fish welfare guaranteed label’ (Solgaard et al., 2023), while another study found that informing German ‘consumers specifically about animal welfare consequences associated with the organic label significantly increases the likelihood of choosing the labelled product and increases the marginal willingness to pay €2.4/kg for organic trout’ (Ankamah‐Yeboah et al., 2019). Earlier studies also found that Norwegian consumers were willing to pay more for welfare‐certified farmed salmon (Olesen et al., 2010) and for improved fish health and welfare in breeding programmes (Grimsrud et al., 2013). It is also noteworthy that the studies presented above concerning public perception of fish and willingness to pay for their welfare are about farmed fish and are from Europe. This is likely so because general farm animal welfare is already a big issue in Europe, particularly in the European Union, for example regarding pig production (Sandøe & Christensen, 2024), but also in the UK and Norway.

There are some studies documenting a growing public concern about the negative effect of fisheries on the environment (e.g. Mazur & Curtis, 2019). Moreover, a global review of public attitudes to angling documents that critical, welfare‐oriented discourses are growing but that people generally view recreational angling as an acceptable pastime (Arlinghaus et al., 2021). However, recreational angling has also given rise to controversies (Jacquet et al., 2020). There are also public concerns when animals are used for scientific purposes (see, e.g., McGlacken & Hobson‐West, 2022; Ormandy & Schuppli, 2014) including the use of fish (Ormandy et al., 2012; Thompson‐Iritani et al., 2026), although fish use may generate less public interest than for other species (Message & Greenhough, 2019). When considering public perceptions of animals held in zoos and aquaria, studies focusing on attitudes to aquaria are in the minority (e.g. Villarroya et al., 2024) and other authors have stated that public awareness of fish welfare in home aquaria should be improved (Maia et al., 2025).

### 10.2 Fish welfare: regulatory mechanisms

A spectrum of regulatory mechanisms affects fish welfare, each with attendant priorities, strengths and weaknesses. At one end of the spectrum, legislation by governmental bodies is ideally held accountable by internal oversight, public law and the external public. These laws reflect slow‐to‐change political priorities with obligatory compliance (Bew, 2016). Enforcement is generally poorly funded, with prosecution rates of less than 1% in most developed countries (Manning et al., 2021). At the other end of the spectrum are mechanisms used by certifying bodies and scientific organisations. Corporate social responsibility commitments are increasingly popular, with members accountable to regulators, stakeholders and scientific communities. This mechanism generally relies on obtained funding or investment, is comparatively fast to change and is well‐funded. Membership is voluntary, and enforcement is often centred around withholding or denying membership. However, breaches may also be subject to remedies under private law.

Another category is certification by a recognised authority. The Sustainable Development Goals 14.2 and 14.4 of the United Nations are particularly relevant here (Marine Stewardship Council, 2023). The growing popularity of this category parallels rising ethical consumerism and reflects trust in awarding authorities. However, this trust has been attacked in the public sphere, for example farm welfare concerns led to a Royal Society for the Prevention of Cruelty to Animals Assured food label review (Prior, 2024). Regulations outside these corporate scenarios mainly exist in, and apply to, tourism, wild‐caught and commercially farmed fish. Another instance is when fish and other animals are used for scientific purposes, and this is routinely scrutinised through national or international directives (e.g. Directive 2010/63/EU – Protection of Animals Used for Scientific Purposes, 2010), or scientific publications.

Global and regional standards to follow are fragmented but offered by groups, including the World Organisation for Animal Health (WOAH, previously OIE) and regional European Union (EU) regulations, which regulate fish used for sustenance and for scientific purposes. Several factors contribute to this challenge, notably finding suitable WIs and evaluating how they should be assessed. Most standards here deal with the high‐profile commercial areas of slaughter and transport. Some countries have attempted to codify these standards, but they have received criticism for being too vague and unspecific to enforce effectively (OECD, 2019). In countries with federal and state divides, including the United States and Australia, federal levels typically deal with fisheries, with individual fish welfare being enacted state by state with limited federal or state protection (Nichols, 2018). There has been a recent rise in anti‐shark finning legislation across many states, but few prosecutions. Governmental enforcement faces many challenges, including illegal fishing and local employment interests. Other challenges include limited regulatory knowledge and capacity, but technological advances, including surveillance drones to find illegal fishers and AI‐based approaches to monitoring WIs, show promise.

While individual fish in the UK are provided basic protection by national welfare acts, farmed fish are excluded from more detailed welfare regulations (UFAW, n.d.) with the Fisheries Act of 2020 covering fisheries but not welfare. The Scottish Animal Welfare Commission has recently published a paper on the policy implications of sentience in all fish (Scottish Animal Welfare Commission, 2025). Fish farmed in Norway are farmed under (i) the Food Act (Matloven, LOV‐2003‐12‐19‐124), (ii) the Animal Welfare Act (Dyrevelferdsloven, LOV‐2009‐06‐19‐97) and (iii) the Aquaculture Act (Akvakulturloven, LOV‐2005‐06‐17‐79), which directly or indirectly consider fish health and welfare (see, e.g., Gismervik et al., 2020). In the EU, fishes are recognised as sentient beings by the EU in Article 13 of the Treaty on the Functioning of the European Union. However, there are questions to what extent a ‘farmed’ fish in Europe is currently protected by EU law and if the international animal welfare standards set out by the WOAH are actually met (Giménez‐Candela et al., 2020). Regulatory certifying bodies and organisations like People for the Ethical Treatment of Animals (PETA) are increasingly active in these areas.

The largest‐scale human‐fish welfare challenge facing wild‐caught and farmed fishing globally is unnecessary mortality. There are many factors that can increase the likelihood of mortality events in aquaculture, including increasingly intensive methods with larger farmed populations, pollution or climate‐related extreme events. In commercial fisheries, the welfare of wild bycatch discards and those caught to become fishmeal (about half of the fish caught globally; Mood & Brooke, 2024) is substandard.

The basic needs of pet fish are best protected in countries that legally recognise fish sentience, that is the EU, and by the Animal Welfare Acts of the UK (2006 for care and 2022 for sentience) and New Zealand (1999) but are complicated by comparatively lower veterinary knowledge levels than that of land animals (a challenge across all categories). Outside these regions, care for pet fish differs immensely. Conversely, fish used for scientific purposes are considered in many regulatory arenas. Various directives, for example Directive 2010/63/EU – Protection of Animals Used for Scientific Purposes 2010, are particularly comprehensive, but lack species‐specific information for fish, except for a recent amendment that considers zebrafish specifically (Commission Delegated Directive (EU), 2024/1262 of 13 March 2024 Amending Directive 2010/63/EU, 2024). Research in developed countries, including North America and Australia, is heavily driven by corporate funding and institutional permissions. Large populations, controlled environments, scrutinised findings and a strong drive for optimal outcomes contribute to an increased awareness of fish welfare. Many of these benefits have migrated to other categories of human–fish interactions.

Awareness of fish welfare in the recreational fisheries sector has been somewhat overshadowed by ongoing fish pain disputes (Jacquet et al., 2020) and, in Germany, has become sidetracked by catch‐and‐release debates (Eckhardt, 2024). While anglers in Norway assist commercial farmers by identifying escaped farmed fish, and ethical practices are lauded on many angling and trophy fishing websites, winners of trophies can also receive purses in the millions for landing fish that ‘fight back’, a practice which directly opposes commitments to reduce suffering in other categories. Some social movements focus on addressing fish welfare issues (e.g. KeepFishWet.org), and extensive education programs by NGOs, the fishing industry and government natural resource management agencies involve recruiting and educating the next generation of anglers. Most natural resource management agencies focus on ensuring that recreational fisheries are sustainable and responsible (Cooke et al., 2019).

Public aquaria have historically been criticised for unnatural environments and separating breeding populations from each other and wild populations. However, they have recently developed a more substantial conservation and welfare focus. The Association of Zoos and Aquariums noted their focus had moved from groups to increasing longevity, supporting breeding programs (Treasure‐Smith, 2025) and WIs.

In closing, if inherent conflicts are managed, fish welfare can benefit from more holistic thinking by better‐trained professionals seeking win/win outcomes. There is also guidance on the best combinations of regulatory mechanisms to influence human behaviour positively (Michie et al., 2011). On a global scale, there is a wide range of regulatory options, responses and challenges to improve human–fish interactions. Whilst we have primarily considered different types of human–fish interaction through a geographical lens, social and economic factors are highly influential and worthy of further review and research. A positive example is CEREBAL, a participatory project that aims to establish a reference centre for fish welfare in Latin America and the Caribbean. There are 28 participating institutions, 17 of which are from the Caribbean and Latin American countries and include governmental departments, universities and NGOs (https://bienestaranimal.unam.mx/).

## 11 KNOWLEDGE GAPS AND FUTURE DIRECTIONS TO EFFECTIVELY IMPROVE FISH WELFARE

**FIGURE 4:**
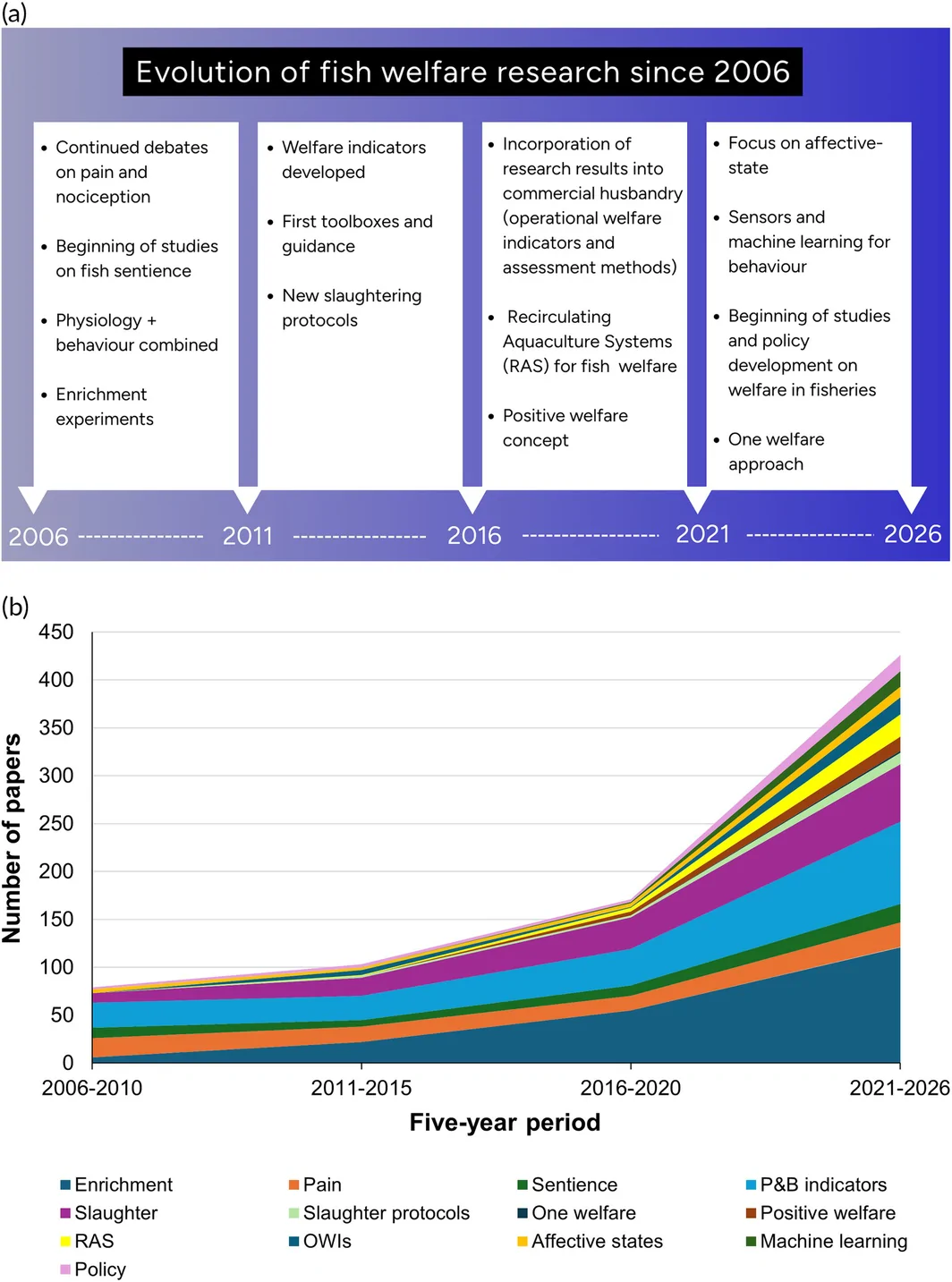
(a) A simplified overview highlighting how fish welfare research has changed and evolved over the last 20 years using the search term ‘fish welfare’, explicitly acknowledging that some research sources are available from earlier years that do not use our specific search terminology. (b) The specific number of papers resulting from the search related to the knowledge gaps and future directions of fish welfare research over the last 20 years. Web of Science was used as a reliable and curated database search engine and a range of search terms combined with the search term ‘fish welfare’ was used per year to download all the literature available. Other related search terms used were ‘environmental enrichment’ and ‘fish welfare’, ‘pain’ and ‘fish welfare’, ‘fish’ and ‘sentience’ and ‘welfare’, ‘physiology’ and ‘behaviour’ and ‘fish welfare’, ‘slaughter’ and ‘fish welfare’, ‘slaughter’ and ‘protocols’ and ‘fish welfare’, ‘one welfare’ and ‘fish’, ‘positive welfare’ and ‘fish’, ‘recirculating aquaculture system’ and ‘fish welfare’, ‘operational welfare indicator’ and ‘fish welfare’, ‘affective states’ and ‘fish welfare’, ‘machine learning’ and ‘fish welfare’, and ‘policy’ and ‘fish welfare’. Additional terms included ‘aquaculture’, ‘ornamental’, ‘farmed’ and ‘aquarium’.

We understand from this paper that, although our knowledge of fish welfare has grown in the last 20 years, some areas still need considerable attention (see Figure 4, which summarises some key topics in fish welfare research over the last 20 years). Here, we highlight the main gaps and some directions for safeguarding and ensuring fish welfare.

### 11.1 Pain in fish

We recognise that much research remains to be done on the mechanisms and consequences of pain in fish and that there remain diverse perspectives among the scientific community about the quality and quantity of evidence regarding the fish pain debate (e.g. Diggles et al., 2023). Indeed, even within the authorship team of this paper there are rather disparate perspectives ranging from strong scepticism to full embracement of the concept. As reviewed in the section on fish neurobiology, the hypothesis asserting fish can feel pain is supported by a growing body of solid scientific evidence convergent from various sources. For some authors, however, whether fish have the capacity to feel pain or not is rather independent of the collective interest in studying fish welfare and can be done without invoking the fish pain debate. But given the substantial neurobiological and behavioural evidence indicating that fish likely have subjective experience and may experience pain, others feel that recognising and considering their capacity to suffer is necessarily central and paramount to fish welfare.

### 11.2 Cognition and affective states

Studies regarding fish cognitive ability and affective states have grown in the last 20 years, therefore giving the notion that fish perceive their own environment and react to it in a complex way. Whereas the approach for welfare for a long time was ‘to be in a state of good welfare is being far from the negative stimuli and stressors’, the modern approach considers positive states of fish welfare. This is a challenge to stakeholders as we face the problem of how to identify such states in fish.

### 11.3 Technology and welfare indicators

The range and diversity of appropriate, fit‐for‐purpose WIs has markedly expanded during the last decade, and steps are being taken to move beyond more basic WIs towards more diagnostic tools. For example, emerging technologies have been created to better monitor and record an array of WIs in fish farming, although many indicators are still a challenge to digitalise in operational environments. In this scenario, WIs may have been studied and are appropriate for documenting the fulfilment of one or more welfare needs, but it is necessary to translate them into technology for specific environments and particular species and life stages, especially as fish comprise thousands of species. Welfare assessment frameworks, based on, for example, semantic modelling, are also increasingly used and available for fish species, which was not the case when the original briefing paper was published in 2006. The use of technological solutions for welfare assessment, as well as the exploitation of AI for improved autonomous assessment of data from cameras, acoustic sensors and implants, is already a reality, particularly in precision fish farming. However, cheaper and accessible technologies are needed to reach a broader set of stakeholders, particularly in developing nations.

### 11.4 Climate change

One of the most evident and challenging problems is climate change, which affects fish welfare in several ways. Although climate change and its effects on animals are shown by current research, its effects on fish welfare, especially in confined environments, are understudied. Effects of climate change also need to be considered for different fish species and, if possible, those more sensitive to environmental changes must be identified.

### 11.5 Wild capture and good fishing practices

Research is needed to improve the welfare of fish caught for both commercial and subsistence fisheries to promote the sustainability of this valuable living resource through improved survival of unwanted catch and by improving meat quality in the landed catch. A good welfare assessment for fisheries is paramount to achieve this goal together with new legislation and policy requirements.

### 11.6 Slaughter practices

One of the ongoing issues regarding fish welfare is how to reduce suffering during slaughter. Some standard practices have been proposed, but they are still developing. Research is still needed, for example, to develop more precise and effective humane slaughter methods, using the guidance of European Food Safety Authority (EFSA) (2018). In the guidance of EFSA (EFSA AHAW Panel, 2018) a two‐step approach is advised: (1) establish a proof of concept using EEGs under controlled laboratory conditions and (2) perform experiments in a commercial setting to verify the effectiveness of the process of stunning and killing. Educative activities to approach stakeholders should also be developed together with legislation changes.

### 11.7 Legislation improvement and implementation

The legal frameworks for safeguarding fish welfare for the different uses of fish should be expanded and built on where needed and effectively implemented. Obviously, these laws can vary according to the country, but efforts could be made to create synergies where appropriate. Ideally, in an era of climate change, One Welfare would be included in such legislation.

### 11.8 Educational activities

Much has been achieved over the last 20 years relating to translating and communicating scientific findings on fish welfare into practice for differing stakeholders in various countries, arenas and operational settings. However, this achievement has not been uniform across different regions and stakeholders. Efforts should be built on and also applied in arenas, sectors or regions where this realisation is less well developed (e.g. some fisheries, some aspects of the ornamental fish trade). Efforts should also be made to sustain and raise public awareness of scientific evidence relating to fish welfare in the context of research, aquaculture, fisheries, aquariums and pastimes (e.g. with regard to the latest findings on neurobiology, emotional states and more welfare friendly ways of handling fish) as public opinion generally affects legislation and habits relating to, for example, their capture, rearing or consumption.

## AUTHOR CONTRIBUTIONS

SRP, EGF and JLS generated the idea of the manuscript and led the funding and manuscript conceptualisation and preparation (overview and contents) as well as writing, editing and preparation. All other co‐authors contributed equally to the manuscript conceptualisation, writing, editing and preparation.

## FUNDING INFORMATION

This review was supported by a grant provided by the Fisheries Society of the British Isles briefing paper scheme (https://fsbi.org.uk/briefing-papers/).

## CONFLICT OF INTEREST STATEMENT

None of the authors have a conflict of interest to disclose.

## Data Availability

Data sharing not applicable to this article as no datasets were generated or analysed during the current study.
